# Effect of Cd on Pyrolysis Velocity and Deoxygenation Characteristics of Rice Straw: Analogized with Cd-Impregnated Representative Biomass Components

**DOI:** 10.3390/ijerph19158953

**Published:** 2022-07-23

**Authors:** Zhi Xu, Zhaohui Guo, Huimin Xie, Yulian Hu

**Affiliations:** Institute of Environmental Engineering, School of Metallurgy and Environment, Central South University, Changsha 410083, China; csuxuzhi@csu.edu.cn (Z.X.); hmxie6@csu.edu.cn (H.X.); 15775813578@163.com (Y.H.)

**Keywords:** rice straw, cadmium, pyrolysis velocity, deoxygenation, biochar

## Abstract

The pyrolysis characteristics of cadmium (Cd)-impregnated cellulose, hemicellulose, and lignin were studied to elucidate the pyrolysis velocity and deoxygenation characteristics of Cd-contaminated rice straw. The results show that Cd significantly affects the pyrolysis characteristics of a single biomass component. With a heating rate of 5 °C·min^−1^ and a Cd loading of 5%, the initial pyrolysis temperature of cellulose and hemicellulose decreases while that of lignin increases. The maximum pyrolysis velocity of cellulose, hemicellulose, and lignin is decreased by 36.6%, 12.4%, and 15.2%, respectively. Cd increases the pyrolysis activation energy of the three components and inhibits their deoxygenation. For the pyrolysis of Cd-contaminated rice straw, both the initial depolymerization temperature and the pyrolysis velocity of hemicellulose is reduced, while the pyrolysis velocity of cellulose is accordingly increased. When Cd loading amplifies to 0.1%, 1%, and 5%, the maximum pyrolysis velocity of hemicellulose is decreased by 7.2%, 10.5%, and 21.3%, while that of cellulose is increased by 8.4%, 62.1%, and 97.3%, respectively. Cd reduces the release of volatiles and gas from rice straw, such as CO_2_, CO, and oxygen-containing organics, which retains more oxygen and components in the solid fraction. This research suggested that Cd retards the pyrolysis velocity and deoxygenation of rice straw, being therefore beneficial to obtaining more biochar.

## 1. Introduction

Heavy-metal-contaminated biomass (HMCB), a solid waste with potential environmental risks, is produced in the process of crop cultivation on contaminated soil, the harvest of phytoremediation engineering, and the adsorption treatment of wastewater by biomass materials [1,2,3,4]. In China, soil heavy metal pollution is serious [5]. Notably, Cd is the principal inorganic contaminant in cultivated soil and as a result, large amounts of Cd-contaminated straw crops are generated, such as Cd-contaminated rice straw [6]. This Cd-contaminated rice straw cannot be reused as fertilizer and feed or privately burned. Therefore, finding a method to dispose of the Cd-contaminated rice straw with suitable technologies is urgent [7].

Pyrolysis is regarded as a promising technology for the treatment of HMCB [8], which can convert various types of lignocellulosic biomass into higher-value-added biochar, bio-oil, and biogas [9,10]. Furthermore, pyrolysis treatment enriches the original heavy metals in biochar manyfold, being conducive to subsequent resource recovery [11,12,13]. The original heavy metals in biomass can be changed from an active state to a relatively stable state through pyrolysis treatment, thus reducing the environmental risk for biochar reuse [11,14]. For example, the heavy metals in contaminated biomass were changed from an acid-soluble/exchangeable state to a residual state after pyrolysis treatment, and the bioavailability of heavy metals was also significantly reduced [15].

However, the effect of concentrated heavy metal elements on biomass pyrolysis could be easily overlooked. Indeed, it was universally found that the heavy metal elements would act as a heat medium and catalyst during the process of biomass pyrolysis. Cu could reduce the pyrolysis activation energy [16,17] and increase the yield of biochar and bio-oil [18]. Pb could enhance the generation of bio-oil [19] and inhibit the pyrolysis of the cellulose component [20]. Zn could enhance the pyrolysis of the cellulose component [20], promote the quality of bio-oil [21,22], cleave oxygen-containing bonds, and reduce the pyrolysis temperature for biomass decomposition [23,24]. These results indicate that heavy metal elements changed the difficulty of biomass pyrolysis and the distribution of pyrolysis products. Of course, the above studies were often carried out by artificially introducing concentrated heavy metal elements into biomass. Nzihou et al. [25] systematically reviewed the effects of inherent and adsorbed metals on biomass pyrolysis, showing that the preparation of heavy-metals-concentrated biomass by artificial addition is feasible. Even so, it is currently unknown whether exogenetic Cd possesses the ability to influence the pyrolysis process of biomass and what influence Cd can provide, which is the research basis for revealing the effect of endogenous Cd on biomass pyrolysis. Therefore, the following two points need to be addressed to fill the gap: Firstly, it is well-known that the pyrolysis of lignocellulosic biomass is usually regarded as a global reaction. The pyrolysis characteristics of lignocellulose are extremely complex that contain many parallel reactions involving the pyrolysis of cellulose, hemicellulose, and the lignin component. The effects of Cd on the pyrolysis of different components may vary significantly. Therefore, the influence mechanism of Cd on the individual pyrolysis behavior of the cellulose, hemicellulose, and the lignin component should be preliminarily revealed, including thermogravimetric characteristics, pyrolysis kinetics, etc. Secondly, theoretical research is more meaningful when it guides actual production. Assuming that the presence of Cd is beneficial to the pyrolysis of rice straw, it can be further concluded that it is wise to use the pyrolysis method for the disposal of Cd-contaminated rice straw. So, the influence mechanism of Cd on the individual pyrolysis of three model components should be verified or applied to the natural rice straw’s pyrolysis treatment.

Therefore, this study reveals the pyrolysis characteristics of three Cd-impregnated biomass components, including cellulose, hemicellulose, and lignin. The thermogravimetric behavior, devolatilization performance, pyrolysis kinetics, and pyrolysis residue characterization of the three representative components were studied in detail. Then, the pyrolysis characteristics and kinetics of Cd-contaminated rice straw were studied to verify the pyrolysis principle obtained from the model components. Additionally, thermogravimetry coupled with Fourier transform infrared spectrometry (TG-FTIR) and thermogravimetry coupled with mass spectrometry (TG-MS) were used to elucidate the release performance of the volatile product from Cd-contaminated rice straw. This study aims to provide new insights into the pyrolysis treatment of Cd-contaminated rice straw.

## 2. Materials and Methods

### 2.1. Preparation of Cd-Impregnated Biomass Components and Rice Straw

The model cellulose (CAS number: 9004-34-6, CE), xylan (CAS number: 9014-63-5, XY), and lignin (CAS number: 9005-53-2, LG) were purchased from Shanghai Macklin Biochemical Co. Ltd., China. The xylan was selected to represent hemicellulose. The Cd-contaminated rice straw (RS) was collected from an experimental base in Hunan province, China. The original rice straw was digested using HNO_3_-HClO_4_ (*v*:*v* = 5:1) to determine the Cd content in rice straw by inductively coupled plasma-atomic emission spectrometry (ICP-AES, ICAP 7400, Thermo Fisher, Waltham, MA, USA). Table 1 lists the basic properties of three model biomass components and rice straw. The content of cellulose, hemicellulose, and lignin in rice straw is about 39.7%, 24.8%, and 18.5%, respectively, which is shown in Table S1.

A Cd loading of 5% (wt.%) was selected as a concentrated level to depict clearer influences of Cd in the process of biomass pyrolysis. The solution impregnation method was applied for the preparation of Cd-concentrated samples according to Fan et al. [26]. Specifically, the corresponding weight of the model biomass component and cadmium chloride (CdCl_2_·2.5H_2_O, analytical purity ≥ 99.0%) was weighed in a beaker, and then plenty of de-ionized water was added for the complete soak. After being stirred for 6 h, the mixture was freeze-dried in a vacuum lyophilizer to remove the moisture. All control samples were also soaked in deionized water without Cd impregnation, stirred, and freeze-dried.

Based on the original contaminated RS (Cd content of about 0.001%), the impregnated RS with a Cd loading of 0.1%, 1%, and 5% was also prepared by the same solution impregnation method. Therefore, four kinds of RS samples with different Cd loadings were obtained to study the effect of Cd on the pyrolysis behavior of RS. The measured content of Cd in Cd-impregnated samples was shown in Table S2. The Cd-concentrated cellulose, xylan, and lignin were named CE-5%Cd, XY-5%Cd, and LG-5%Cd, respectively. The RS-0.1%Cd, RS-1%Cd, and RS-5%Cd represented the rice straw with Cd loading of 0.1%, 1%, and 5%, respectively.

### 2.2. Thermogravimetric Analysis and Pyrolysis Experiments

The thermogravimetric analysis (TG) and differential thermogravimetric analysis (DTG) were applied with a thermal analyzer (TGA 8000, PerkinElmer, Waltham, MA, USA) connected to a gas flow system to work in the N_2_ atmosphere (20 mL·min^−1^). For each test, about 10 ± 0.5 mg of the sample were employed. Four different heating strategies (5, 10, 20, and 50 °C·min^−1^) were adopted to collect the thermogravimetric data from room temperature to 700 °C. The pyrolysis TG/DTG curves of CdCl_2_·2.5H_2_O are shown in Figure S1.

The fixed-bed pyrolysis experiments in a tube furnace were conducted with the N_2_ atmosphere (20 mL·min^−1^). The sample was placed in a quartz crucible and heated to 700 °C with a heating rate of 5 °C·min^−1^. After cooling to room temperature, the pyrolysis residue was collected for characterization.

### 2.3. TG-FTIR and TG-MS Experiments for Cd-Contaminated RS

For the TG-FTIR experiments of Cd-contaminated RS, the functional group characteristics of pyrolytic volatiles were analyzed using a thermogravimetric analyzer (TGA 8000, PerkinElmer, Waltham, MA, USA) combined with a Fourier transform infrared spectrometer (Frontier, PerkinElmer, Waltham, MA, USA). In the N_2_ atmosphere (20 mL·min^−1^), the rice straw samples were heated from room temperature to 700 °C at a heating rate of 20 °C·min^−1^. The generated volatile products were transmitted to an infrared spectrometer through a transmission line for real-time online analysis. The temperature of the transmission line was set at 300 °C to prevent the condensation of volatiles. The scanning range of the infrared spectrum was 500–4000 cm^−1^ with a resolution of 4 cm^−1^. The background interference has been deducted before the experiment.

For the TG-MS experiments of Cd-contaminated RS, the release characteristics of pyrolytic gas products were analyzed using the thermogravimetric analyzer (SDT 650, TA Instruments, USA) combined with a mass spectrometer (Discovery MS, TA Instruments, New Castle, DE, USA). In the N_2_ atmosphere (20 mL·min^−1^), the rice straw samples were heated from room temperature to 600 °C at a heating rate of 20 °C·min^−1^, and the gas products were transferred to the mass spectrometry analyzer for real-time online analysis. The temperature of the transmission line was set at 300 °C to prevent water vapor condensation. The ion source energy of the mass spectrometer was 70 eV, and the molecular weight range of the mass spectrometry was 1–300.

### 2.4. Kinetic Analysis Based on Isoconversional Method

The overall rate, *dα/dt*, of a solid-state reaction is expressed in the following form [27]:

(1) $$\frac{d\alpha }{dt}=\beta \frac{d\alpha }{dT}=Aexp\left(-\frac{E}{RT}\right)f\left(\alpha \right)\;$$

where *α* represents the pyrolysis conversion rate; *t* represents the pyrolysis time (min); *β* represents the heating rate (K·min^−1^); *T* represents the reaction temperature (K); *A* represents the pre-exponential factor (min^−1^); *E* represents the activation energy (kJ·mol^−1^); *R* is the universal gas constant (8.314 J·mol^−1^·K^−1^); *f*(*α*) accounts for reaction rate dependence on α.

The conversion rate (*α*) of biomass pyrolysis can be given in the following form:

(2) $$\alpha =\frac{w_{0}-w_{t}}{w_{0}-w_{f}}\;$$

where *w*_0_ represents the mass of the initial sample; *w_f_* represents the residual mass of the sample at 700 °C; *w_t_* represents the mass at an instant *t*.

The isoconversional method includes differential form and integral form [28]. The most common differential form is the Friedman method, which requires high precision of the thermal analyzer and data. By taking a logarithm of Equation (1), the Friedman method can be expressed in the following form:

(3) $$\ln\left(\beta \frac{d\alpha }{dT}\right)=\ln\left[Af\left(\alpha \right)\right]-\frac{E}{RT}\;$$

For a given conversion rate, the apparent pyrolysis activation energy (APAE) can be calculated from the linear relation between *ln*(*βdα/dT*) and 1/*T* according to Equation (3).

The most common integral forms include the Flynn–Wall–Ozawa (FWO) method and the Kissinger–Akahira–Sunose (KAS) method. The FWO method can be expressed in the following form [29]:

(4) $$\ln\left(\beta \right)=\ln\left[\frac{AE}{Rg\left(\alpha \right)}\right]-5.331-1.052\frac{E}{RT}$$

For a given conversion rate, the APAE can be calculated from the linear relation between *ln*(*β*) and 1/*T* according to Equation (4).

The KAS method can be expressed in the following form [30]:

(5) $$\ln\left(\frac{\beta }{T^{2}}\right)=\ln\left(\frac{AR}{Eg\left(\alpha \right)}\right)-\frac{E}{RT}\;$$

Similarly, the APAE can be calculated from the linear relation between *ln*(*β*/*T*^2^) and 1/*T* according to Equation (5).

More details about fitting results by the isoconversional methods can be seen in Table S3.

### 2.5. Characterization of Pyrolysis Residues

The three model components and the corresponding pyrolysis residue were analyzed by Fourier transform infrared spectrometry (FTIR, Nicolet IS10, Thermo Fisher, Waltham, MA, USA), X-ray photoelectron spectroscopy (XPS, K-Alpha X, Thermo Fisher, Waltham, MA, USA), and elemental analyzer (Elementar Vario III, Thermo Fisher, Waltham, MA, USA). For FTIR analysis, the mass ratio of sample to KBr was about 1:100 and the scan range was from 500 to 4000 cm^−1^. For XPS analysis, the Al Kα excitation source was used. Before analyzing the data of XPS, all the samples were calibrated based on C 1 s with a binding energy of 284.8 eV.

## 3. Results and Discussion

### 3.1. Pyrolysis Characteristics of Cd-Concentrated Cellulose, Hemicellulose, and Lignin

#### 3.1.1. TG/DTG Analysis

**Figure 1:**
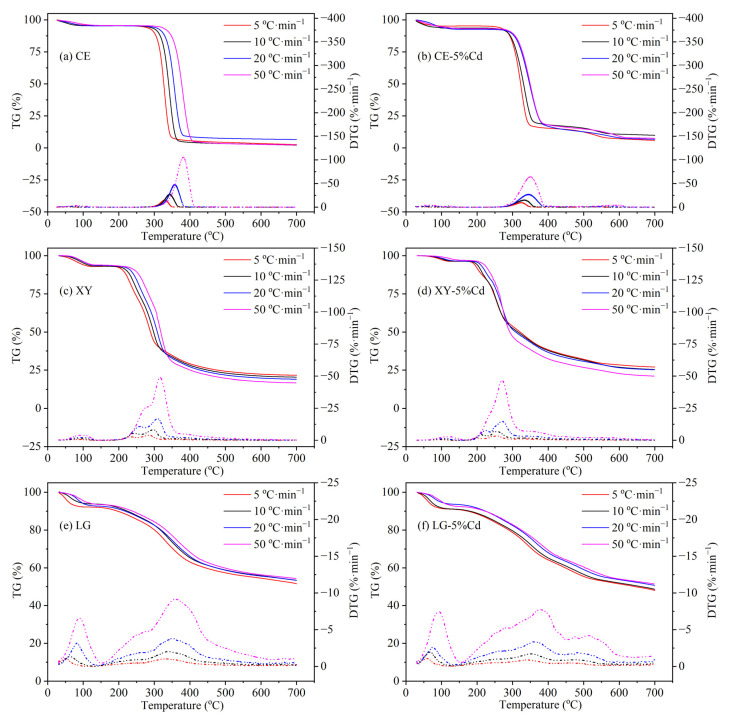
TG/DTG curves of three model components at different heating rates. (**a**,**c**,**e**) are the control groups; (**b**,**d**,**f**) are the Cd-concentrated biomass with a Cd loading of 5% (wt.%).

The TG and DTG curves of the three model components and their Cd-impregnated samples are shown in Figure 1. The pyrolysis processes of the three biomass components exhibit three stages including dehydration, rapid decomposition with significant weight loss, and slow carbonization resulting in biochar as a solid product. Taking the TG and DTG curves at 5 °C·min^−1^ for instance, there is no obvious mass loss of cellulose before 280 °C except for the initial dehydration process (Figure 1a). The main pyrolysis region of cellulose is in the temperature range of 280–350 °C. The maximum pyrolysis velocity of cellulose is reached at 329 °C with 14.9 %·min^−1^. After 350 °C, the pyrolysis of cellulose is basically completed and the remaining solid only consists of about 2.3%. According to the Broido–Shafizadeh model [31,32], cellulose is first translated into active cellulose, followed by a fast mass loss through two paths: (1) releasing amounts of volatiles in which levoglucosan is the main compound; (2) generating small-molecule gas by constant depolymerization. Therefore, the pyrolysis products of cellulose are mainly volatiles as well as gas, thus generating fewer solid residues than that of xylan and lignin. Compared with cellulose, xylan possesses poorer thermal stability that starts to crack at 200 °C and reaches the peak of weight loss rate (4%·min^−1^) at 278 °C (Figure 1c). It is obvious that there is a shoulder peak at about 234 °C in the DTG curve of xylan, indicating a two-stage reaction in the pyrolysis process of xylan [33]. The branches of xylan firstly tend to depart from the backbone due to their poor thermal stability, and then, the trunk of xylan begins to depolymerize and rearrange [34,35,36]. Therefore, the two peaks of xylan’s DTG curves may correspond to the two-step reaction. For lignin (Figure 1e), its pyrolysis reaction exhibits the widest temperature range, from 150 to 700 °C. Between 200 and 500 °C, about 30% of mass loss and an obvious peak can be found according to the DTG curves. The mass loss of lignin at this stage is mainly attributed to dehydration, the fracture of the ether bond, and the cleavage of the C–O bond which links the phenol ring and side-chain [37]. The molecular network of lignin starts to split, and gas molecular begins to release at the temperature of 160 °C. Between 200 and 330 °C, the cracking reaction of lignin accelerates to generate heavy and light bio-oil. As the pyrolysis temperature is over 330 °C, more oxygen-containing function groups crack [37]. Therefore, the peak of the lignin’s DTG curve at 350 °C is associated with the oxygen-containing function group’s cracking reaction.

The pyrolysis behavior of the three Cd-concentrated model components is obviously changed. As shown in Figure 1b, the TG/DTG curves of Cd-concentrated cellulose are shifted to the low-temperature region. Taking 5 °C·min^−1^ for instance, the *T_s_* (the starting temperature of pyrolysis) is decreased from 282 to 267 °C and the *T_m_* (the temperature of maximum pyrolysis velocity) is decreased from 329 to 325 °C, indicating that Cd promotes the initial pyrolysis of cellulose at low temperature. Similar results reported by Mayer et al. [20] demonstrated that Zn^2+^ and Fe^3+^ also catalyzed the cellulose degradation with a decreased pyrolysis temperature of 4–9 °C, while Ca^2+^ and Pb^2+^ inhibited the cellulose degradation with an increased pyrolysis temperature of 1–4 °C. Importantly, the above movement trend caused by Cd is more pronounced at the higher heating rate conditions, which indicates that Cd acts as a heat medium during the pyrolysis reaction and greatly reduces the thermal hysteresis effect by increasing the heating rate [38]. In addition, Cd also reduces the peak value of the DTG curve of which *R_m_* (the maximum pyrolysis velocity) is decreased from 14.9 to 9.44%·min^−1^. Generally, active cellulose is first formed by reducing the polymerization degree of cellulose, and then generates levoglucosan and furan through depolymerization, or generates various small molecules of aldehydes and ketones through ring-opening reactions [39]. According to the left shift of TG and DTG curves, Cd may promote the formation of active cellulose, while the decrease of *R_m_* indicates that Cd may inhibit the subsequent decomposition reactions. For Cd-concentrated xylan (Figure 1d), the TG and DTG curves also move to the low-temperature regions and the *R_m_* value is declined slightly. Since the reaction pathways of xylan are similar to cellulose, it can be speculated that Cd also enhances the initial depolymerization of xylan and retards the subsequent decomposition reactions. Conversely, Cd restricts the pyrolysis reaction of lignin that both *T_s_* and *T_m_* are increased from 151 and 331 °C to 166 and 342 °C, and *R_m_* is decreased from 1.05 to 0.89%·min^−1^ (Figure 1f). These results suggest that the presence of Cd is not conducive to the fragmentation of lignin and the cracking of its oxygen-containing functional groups [37]. The above analysis shows that Cd does affect the three model components’ pyrolysis behavior. In general, Cd has a relatively consistent effect on cellulose and xylan that promotes the initial depolymerization but slows down the subsequent cracking. However, the effect of Cd on lignin pyrolysis was mostly negative, increasing the initial pyrolysis temperature and decreasing the maximum pyrolysis velocity.

#### 3.1.2. Devolatilization Performance

The devolatilization performances (*D_i_*) of three model components are further calculated using the following Equation (6) [40]:

(6) $$D_{i}=\frac{R_{m}}{T_{s}T_{m}\Delta T_{1/2}}$$

where *R_m_* represents the maximum pyrolysis velocity; *T_s_* represents the starting temperature of pyrolysis; *T*_m_ represents the temperature corresponding to *R_m_*; Δ*T*_1/2_ represents the temperature difference between *T*_1/2_ and *T_m_*. *T*_1/2_ is the temperature corresponding to the 0.5 *R_m_*.

**Figure 2:**
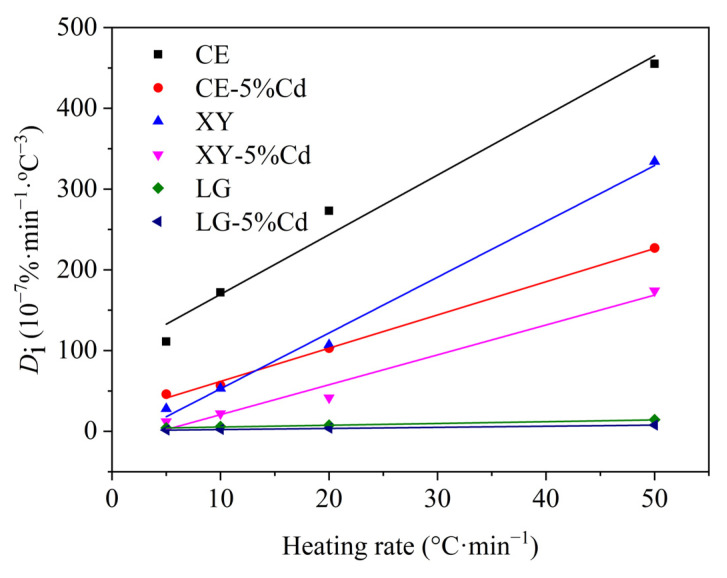
Devolatilization index (*D_i_*) for three model components and their Cd-concentrated samples.

As shown in Figure 2, the *D_i_* value of three model components follows the order of cellulose > xylan > lignin, indicating that cellulose possesses a higher releasing performance of volatiles, followed by xylan and lignin. The stronger devolatilization performance of cellulose is also the reason why the pyrolysis residue from cellulose is less than that of xylan and lignin. Interestingly, the *D_i_* value of three model components is reduced with the involvement of Cd, suggesting that Cd inhibits the devolatilization performance of cellulose, xylan, and lignin. Namely, Cd can promote the formation of solid residue in the view of mass conservation. This seems to explain why the pyrolysis rate of Cd-concentrated cellulose, xylan, and lignin is reduced in Figure 1. Meanwhile, increasing the heating rate promotes the *D_i_* values, indicating that the three model components are more volatile with a high heating rate. Generally, a rapid heating rate shortens the residence time in the low-temperature region and thus equivalently lengthens the residence time in the high-temperature region [40]. Moreover, the coking reaction for char is exothermic while the devolatilization reaction is endothermic, thus a high heating rate promotes volatile release and inhibits the carbonization reaction [40]. Furthermore, the linear relation between *D_i_* values and heating rates shows a sensitivity of devolatilization to heating rate, and the sensitivity follows the sequence of CE > XY > CE-5%Cd > XY-5%Cd > LG > LG-5%Cd, indicating that Cd reduces this sensitivity of cellulose, xylan, and lignin to heating rate, and even inhibits their devolatilization performance at a higher heating rate. The results also suggest that the involvement of Cd is more conducive to the coking of biomass into biochar. In summary, Cd inhibits the volatiles escape of cellulose, xylan, and lignin, and enhances the formation of biochar, which is significantly different from the alkali or alkaline earth metals that catalyze the pyrolysis of biomass to obtain more bio-oil products. Although the pyrolysis characteristics analysis by TG/DTG can help grasp the effect of Cd contamination on macroscopic phenomena of the pyrolysis of cellulose, xylan, and lignin, pyrolysis kinetics analysis needs to be carried out to reveal the inner influence mechanism of Cd.

#### 3.1.3. Pyrolysis Kinetics Analysis

To facilitate the analysis of the change of APAE in pyrolysis progress, the main pyrolysis process with the α between 0.2 and 0.8 is extracted to describe whether Cd also inhibits the kinetic process of the three model components. The corresponding relationship between α and the pyrolysis temperature is supplemented in Table S4. As shown in Figure S2, the Arrhenius plots were calculated using three isoconversional methods. Given fitting results (Table S3), the KAS method and the FWO method are more suitable than the Friedman method, which could be caused by the experimental data noise and numerical instability [41].

**Figure 3:**
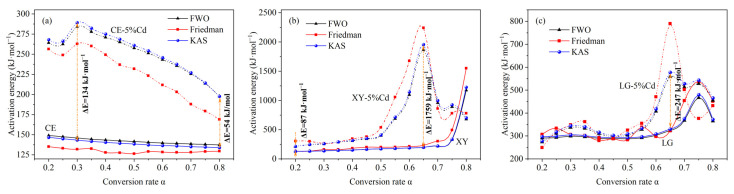
APAE distribution of three model components at the different conversion rates. (**a**) CE and CE-5%Cd; (**b**) XY and XY-5%Cd; (**c**) LG and LG-5%Cd. The solid line and dotted line represent the control group and Cd-concentrated samples, respectively.

The APAE of the three model components with different α is shown in Figure 3. Similar calculation results and variation trends are obtained using the three isoconversional methods. Among them, the results based on the FWO method and the KAS method are highly consistent, indicating that the FWO and KAS methods are more applicable. For cellulose (Figure 3a), the APAE shows a gradual downward trend from 147 to 139 kJ·mol^−1^, which may be due to the lower energy required for the scission of active cellulose [40]. Zong et al. [40] also reported that the APAE of cellulose gradually decreased, with an average of 157 kJ·mol^−1^. When Cd is involved in the pyrolysis reaction, the APAE of Cd-concentrated cellulose is significantly higher than that of cellulose with an increased value of 54–134 kJ·mol^−1^, indicating that Cd increases the devolatilization difficulty of cellulose and thus more energy is needed to maintain the pyrolysis reaction during the cracking process. Generally, the lower APAE represents the faster pyrolysis velocity. Therefore, the reduced pyrolysis velocity of Cd-concentrated cellulose in Figure 1b can be reasonably explained. For xylan (Figure 3b), the APAE is gradually increased from 128 to 221 kJ·mol^−1^ when *α* is increased from 0.2 to 0.7, while a significant increase is observed when *α* is increased from 0.7 to 0.8. The rapid increase of APAE indicates that the devolatilization of xylan was basically completed and that it had entered the slow carbonization stage [40]. For Cd-concentrated xylan, it can be seen that the APAE between *α* = 0.5 and *α* = 0.65 is significantly elevated, indicating that the devolatilization process of xylan is inhibited. Combined with DTG curves of XY and XY-Cd, the inhibited devolatilization process may be the second characteristic reaction of xylan, including the depolymerization and rearrangement of xylan’s trunk [34,35]. As shown in Figure 3c, the APAE of lignin is gradually increased from 297 to 480 kJ·mol^−1^ when *α* is increased from 0.2 to 0.75, indicating that the devolatilization of lignin is poorer than that of cellulose and xylan. Similar to cellulose and xylan, Cd elevates the lignin’s APAE in each pyrolysis stage, especially between α = 0.6 and α = 0.7, indicating that Cd also inhibits the major pyrolysis processes such as fragmentation and bond cracking. In summary, Cd retards the pyrolysis velocity of cellulose, xylan, and lignin by increasing their APAE.

#### 3.1.4. Characteristics of Pyrolysis Residues

**Figure 4:**
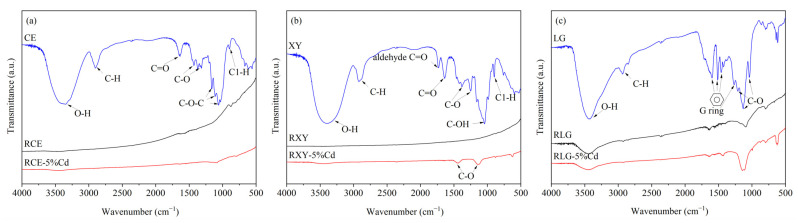
FTIR spectrums of three model components and their pyrolysis residue. (**a**) CE, RCE, and RCE-5%Cd; (**b**) XY, RXY, and RXY-5%Cd; (**c**) LG, RLG, and RLG-5%Cd. RCE, RXY, and RLG represent the pyrolysis residue of cellulose, xylan, and lignin, respectively. RCE-5%Cd, RXY-5%Cd, and RLG-5%Cd represent the pyrolysis residue of Cd-concentrated cellulose, xylan, and lignin, respectively.

As shown in Figure 4, FTIR analysis was further used to verify whether Cd affects the function group characteristics of pyrolysis residues. For cellulose (Figure 4a), the typical characteristic peaks of O–H (3470 cm^−1^), C=O (1640 cm^−1^), C–O (1310–1428 cm^−1^), and C–O–C (1060–1160 cm^−1^) groups disappear after pyrolysis treatment, indicating that strong deoxidation reactions occur during the pyrolysis process, such as dehydration, decarbonylation, and decarboxylic group [42]. After involvement with Cd, the absorption intensity of these oxygen-containing functional groups is slightly increased, indicating that Cd may inhibit the deoxidation reaction of cellulose. Similarly, the peak strength of the C=O (1730 and 1640 cm^−1^) and C–O (1040–1380 cm^−1^) of xylan decreases after pyrolysis, and the presence of Cd also increases the absorption intensity of the C–O group. As shown in Figure 4c, Cd significantly increases the absorption intensity of the C–O group in the pyrolysis residue of lignin, indicating that Cd may inhibit the crack of the C–O group such as the methoxide on the benzene ring. Therefore, a preliminary conjecture may be that Cd could retard the deoxidation reaction of three model components.

**Figure 5:**
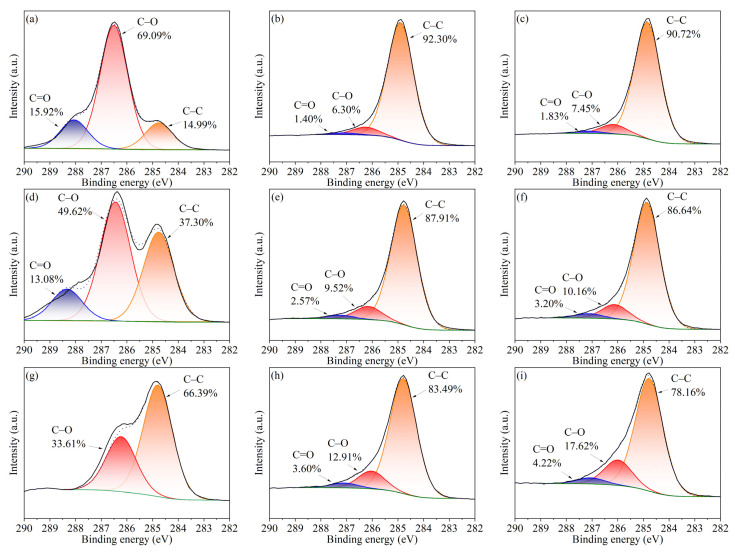
XPS spectra (C1 s) of three model components and their pyrolysis residue. (**a**) CE; (**b**) RCE; (**c**) RCE-5%Cd; (**d**) XY; (**e**) RXY; (**f**) RXY-5%Cd; (**g**) LG; (**h**) RLG; (**i**) RLG-5%Cd. RCE, RXY, and RLG represent the pyrolysis residue of cellulose, xylan, and lignin, respectively. RCE-5%Cd, RXY-5%Cd, and RLG-5%Cd represent the pyrolysis residue of Cd-concentrated cellulose, xylan, and lignin, respectively.

As shown in Figure 5, XPS analysis was used to characterize the C oxidation state (C 1s) of pyrolysis residue. It can be seen that the presence of Cd increases the proportion of the oxidation state of C element. The proportion of C–O in Cd-concentrated cellulose, xylan, and lignin is increased from 6.30%, 9.52%, and 12.91% to 7.45%, 10.16%, and 17.62%, respectively. The proportion of C=O in Cd-concentrated cellulose, xylan, and lignin is increased from 1.40%, 2.57%, and 3.60% to 1.83%, 3.20%, and 4.22%, respectively. The results based on XPS analysis confirmed the hypothesis that Cd may inhibit the decarbonylation and decarboxylic process, increasing oxygen-containing groups in pyrolysis residue.

More intuitive results for element content of C, H, and O in pyrolysis residue are shown in Table 2. After pyrolysis, the C content of cellulose and xylan increases significantly from 42.44% and 41.10% to 89.55% and 85.85%, respectively. Inversely, the O content of cellulose and xylan decreases from 51.20% and 52.27% to 7.40% and 11.10%, respectively. For lignin, the pyrolysis residue still contains an O content of 33.25%, indicating that the deoxidation degree of lignin is significantly lower than that of cellulose and xylan. When Cd is involved in the pyrolysis system, the O content of cellulose, xylan, and lignin is increased from 7.40%, 11.10%, and 33.25% to 8.64%, 12.93%, and 33.82%, respectively, indicating that Cd does inhibit the deoxidation reaction of cellulose, xylan, and lignin, and retains more organic oxygen-containing components in pyrolysis residue.

### 3.2. Pyrolysis Characteristics of Cd-Contaminated Rice Straw

#### 3.2.1. TG/DTG Analysis

**Figure 6:**
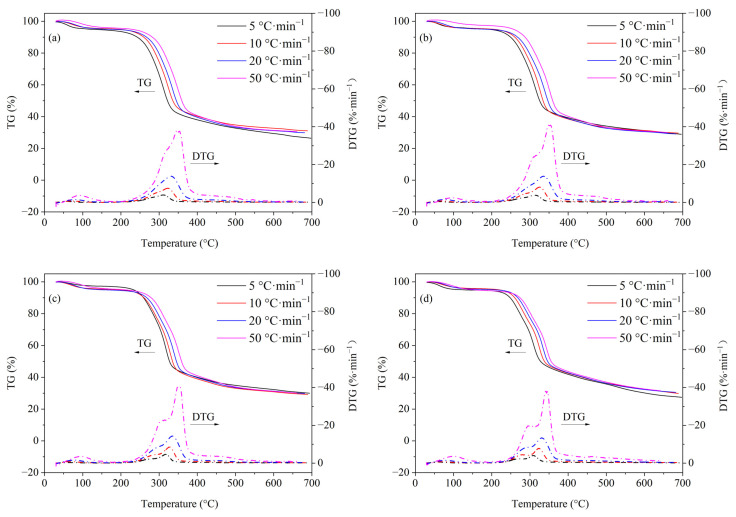
TG and DTG curves of rice straw with different Cd loading. (**a**) RS, (**b**) RS-0.1%Cd, (**c**) RS-1%Cd, and (**d**) RS-5%Cd.

The TG/DTG curves of rice straw with different amounts of Cd loading are shown in Figure 6. At the initial stage of pyrolysis (from room temperature to 150 °C), the rice straw shows a certain weight loss of about 7%, which was mainly related to moisture removal. With the increase of Cd loading, the shape of TG curves does not change significantly and the weight loss rate of four different rice straws is about 70%. However, it can be observed from the DTG curves that Cd has a great influence on the pyrolysis rice straw. Taking 50 °C·min^−1^ for instance, rice straw starts to decompose significantly at 229 °C and then reaches an inapparent peak at 315 °C as well as an apparent peak at 347 °C, indicating that there is a pyrolytic superposition between the hemicellulose component and the cellulose component. The first peak at 315 °C is not salient; that is, it is difficult to observe at the low heating rate. However, as shown in Figure 6b,d, the two DTG peaks of rice straw with Cd loading of 0.1%, 1%, and 5% are easily distinguished, and the separation of the two peaks is more significant with the increase of Cd loading. These results are significantly different from previous studies [8]. Li et al. [8] reported that Zn contamination could increase the overlap between hemicellulose and cellulose. In this study, inversely, Cd contamination promotes the separation between hemicellulose and cellulose. The two opposite effects may be related to the different properties of Zn and Cd. Next, the pyrolysis separation of the cellulose component and hemicellulose component is further studied by using the peak fitting tools in Origin 9.0 software.

**Figure 7:**
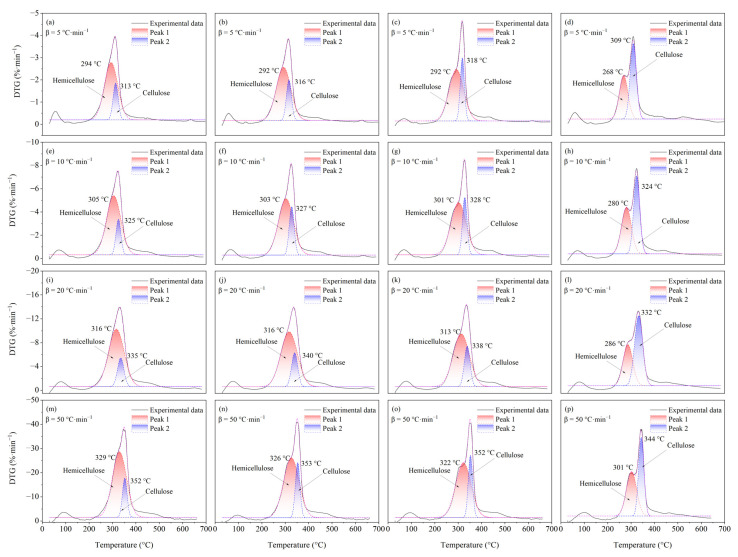
The separate DTG curves of hemicellulose component and cellulose component in rice straw with different Cd loading. (**a**,**e**,**i**,**m**) are the RS; (**b**,**f**,**j**,**n**) are the RS-0.1%Cd; (**c**,**g**,**k**,**o**) are the RS-1%Cd; (**d**,**h**,**l**,**p**) are the RS-5%Cd, respectively.

As shown in Figure 7, the main pyrolysis stage between 200 and 400 °C of DTG curves can be further differentiated into two peaks, which belong to the hemicellulose component (Peak 1) and cellulose component (Peak 2), respectively. First, with the increase of Cd loading, Peak 1 moves to the low-temperature region while the position of Peak 2 is less affected. Secondly, the peak value of Peak 1 is decreased with the increase of Cd loading while that of Peak 2 is significantly increased. Thirdly, the overlap area of Peak 1 and Peak 2 is significantly reduced. Therefore, the above three phenomena indicate that: (1) the presence of Cd promotes the early pyrolysis of rice straw’s hemicellulose component but decreases its pyrolysis rate, which is highly consistent with the results from the pyrolysis of model xylan; (2) as the peak value of the overall DTG curve of rice straw does not change significantly and Peak 1 moves to a low temperature, the contribution of the cellulose component’s pyrolysis to the whole DTG curve of rice straw is significantly increased; (3) the presence of Cd effectively separates the pyrolytic overlap between the hemicellulose component and cellulose component. It is noteworthy that the promoting effect of Cd on the cellulose component of rice straw is significantly different from the model cellulose. For the pyrolysis of model cellulose at a heating rate of 5 °C·min^−1^ (Figure 1a,b), Cd significantly reduces the maximum pyrolysis velocity of cellulose by about 36.6%. However, the maximum pyrolysis velocity of the cellulose component of rice straw is increased by about 11%, 61%, and 100% when the Cd loading is 0.1%, 1%, and 5%, respectively. This contradictory phenomenon may be due to the complexity of the components of rice straw. According to previous studies, the lignin component could catalyze the pyrolysis of the cellulose component [43,44]. Therefore, the cellulose component of rice straw may be catalyzed by the concomitant lignin component, thus increasing the pyrolysis velocity of cellulose. In particular, no separate peaks were subjectively calculated for lignin due to its small contribution to the overall pyrolysis process of rice straw.

#### 3.2.2. Pyrolysis Kinetics Analysis

**Figure 8:**
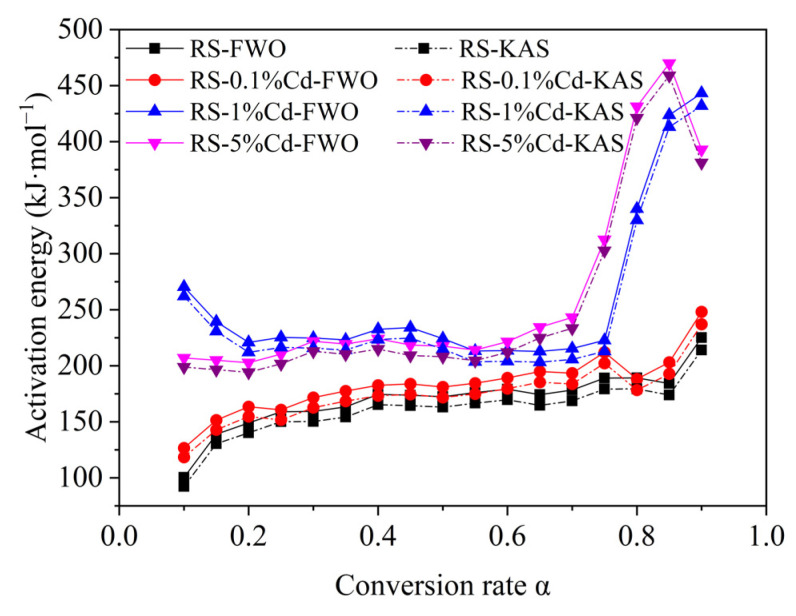
APAE of rice straw with different Cd loading based on FWO method and KAS method.

The APAE of rice straw was calculated based on the FWO method (Figure S3) and the KAS method (Figure S4). According to the fitting results, the early (α = 0.1–0.2) and the late (α = 0.7–0.9) pyrolysis stages have a lower fitting degree than the midterm (α = 0.2–0.7) stage. Therefore, the FWO method and the KAS method can be better applied at the stage of rapid mass loss (α = 0.2–0.7). As shown in Figure 8, the results based on the FWO method and KAS method are highly consistent. During the main pyrolysis stage (α = 0.2–0.7), the APAE of rice straw increases from 148.9 kJ·mol^−1^ to 178.4 kJ·mol^−1^. As Cd loading is gradually increased to 5%, the APAE of rice straw is increased to 202.8–243.2 kJ·mol^−1^, indicating that the presence of Cd increases the APAE of rice straw and thus promotes the difficulty of pyrolysis, which is consistent with the results from the model components.

### 3.3. Characteristics of Pyrolytic Volatiles from Cd-Contaminated Rice Straw

#### 3.3.1. TG-FTIR Analysis

**Figure 9:**
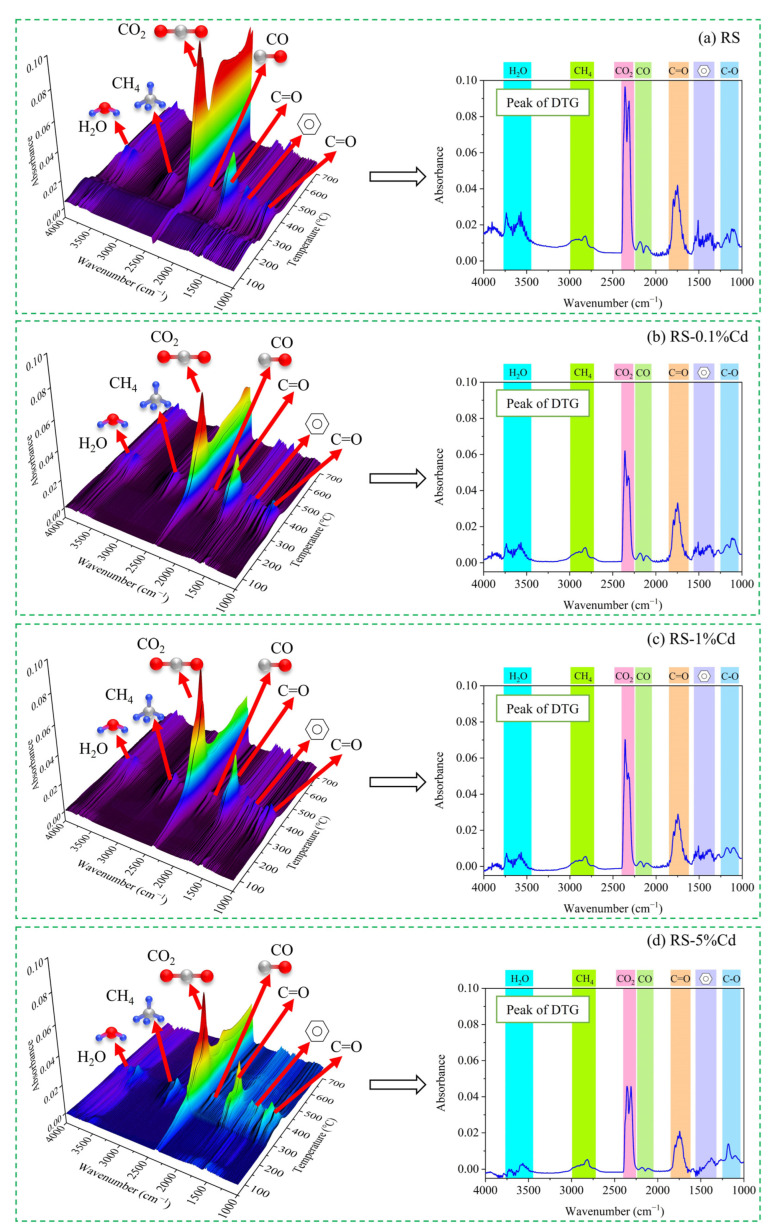
The FTIR spectrum of pyrolytic volatiles from (**a**) RS, (**b**) RS-0.1%Cd, (**c**) RS-1%Cd, and (**d**) RS-5%Cd. The left side is the 3D FTIR spectrums during the whole pyrolysis process at a heating rate of 20 °C·min^−1^. The right side is the extracted spectrums at the temperature corresponding to the DTG curves’ peak.

The infrared spectrum of volatile products from Cd-contaminated rice straw is shown in Figure 9. The temperature region of volatiles releasing is consistent with the DTG curves in the range of 250 °C to 400 °C. The absorption peaks of the main functional groups include the O–H bond (3500–3750 cm^−1^), C–H bond (2780 cm^−1^), CO_2_ (2360 cm^−1^), CO (2190 cm^−1^), C=O bond (1750 cm^−1^), benzene ring (1500 and 1450 cm^−1^), and C–O bond (1100 cm^−1^), indicating that the main detected volatiles include H_2_O, CH_4_, CO_2_, CO, and C=O/C–O/benzene-ring-containing components [40]. The generation of CO_2_ is related to the cracking of carboxyl, carbonyl, and ester groups which are widely found in cellulose, hemicellulose, and lignin components. Similarly, CO is also produced by the cracking of the ether group and carbonyl group. CH_4_ is generated by the cracking of the methoxy group (–O–CH_3_) and methyl group (–CH_3_). The lignin component is the largest contributor to the generation of CH_4_ due to its large amounts of methoxy groups and methyl groups [40,45]. Moreover, the bonds of C=O, C–O, and the benzene ring indicate that the volatiles also includes aldehydes, ketones, acids, and phenolic compounds. In addition, it can be seen on the right side of Figure 9 that Cd affects the generation of volatile products. The absorbance peak of the CO_2_ decreases gradually with the increase of Cd loading. Meanwhile, the absorbance peak of the C=O bond, C–O bond, and CO also declines with the increase of Cd loading. The above results indicate that Cd has an inhibitory effect on the cracking of the oxygen-containing groups in rice straw.

#### 3.3.2. TG-GC/MS Analysis

**Figure 10:**
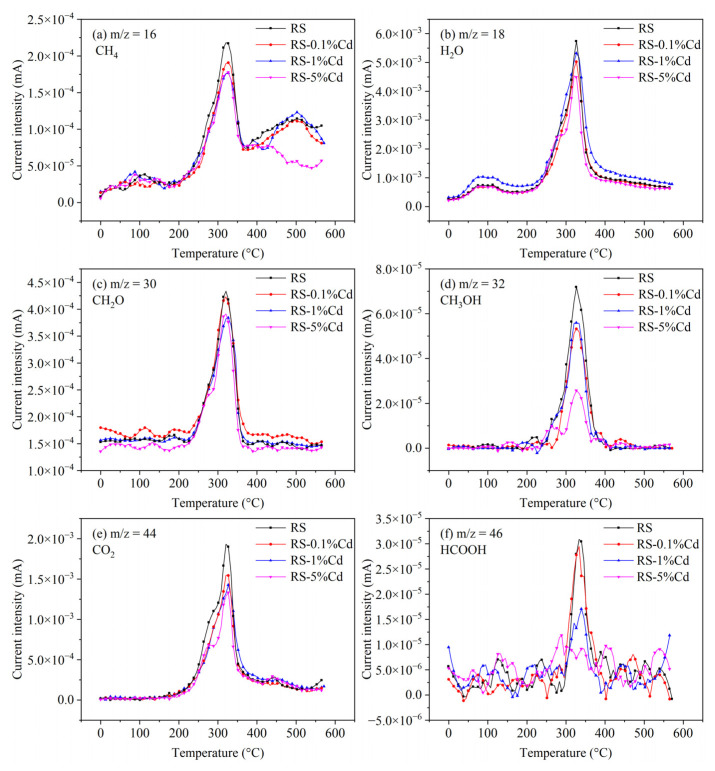
Release characteristics of gaseous products from rice straw with different Cd loading. (**a**) is the CH_4_; (**b**) is the H_2_O; (**c**) is the CH_2_O; (**d**) is the CH_3_OH; (**e**) is the CO_2_; (**f**) is the HCOOH, respectively.

Figure 10 shows the releasing characteristics of gaseous products from rice straw. The gaseous products mainly include CH_4_, H_2_O, CH_2_O, CH_3_OH, CO_2_, and HCOOH. The gaseous products are concentrated in the range of 250 °C to 400 °C which is the pyrolysis temperature of cellulose, hemicellulose, and lignin, indicating that the cellulose component, hemicellulose component, and lignin component are the main sources of gaseous products. For CH_4_ (Figure 10a), it is still generated beyond 400 °C, indicating that CH_4_ is also produced by the latter pyrolysis of the lignin component. With the involvement of Cd in the pyrolysis process of rice straw, the gaseous products are decreased with the increase of Cd loading. For example, as shown in Figure 10e, the yield of CO_2_ is followed by the order of RS > RS-0.1%Cd > RS-1%Cd > RS-5%Cd, which is consistent with the results of TG-FTIR (Figure 9). Similar results are found in Figure 10d,f. The above results verify that Cd provides an inhibition effect on the deoxygenation reaction of rice straw’s pyrolysis. He et al. [12] reported that heavy metals (including Cr, Zn, As, Cd, and Pb) could reduce the yield of pyrolytic gas and bio-oil from *Avicennia marina* biomass but increase the yield of biochar. In this study, the pyrolytic gas and oxygen-containing organic volatiles (Table S5) are reduced by Cd contamination, resulting in the retention of oxygen elements in biochar. Meanwhile, the inhibited deoxygenation of Cd-contaminated rice straw also results in high-quality bio-oil [10]. Therefore, Cd-contaminated rice straw may be more conducive to preparing biochar with high oxygen content and bio-oil with low oxygen content through pyrolysis treatment.

## 4. Conclusions

The effect of Cd on the pyrolysis of lignocellulosic biomass has often been overlooked. The pyrolysis characteristics of Cd-impregnated model cellulose, hemicellulose, and lignin were studied, and the findings from the model components were further verified using the pyrolysis of Cd-contaminated rice straw in this work. The thermogravimetric behavior, pyrolysis kinetics, and properties of pyrolytic products from the Cd-impregnated model components and rice straw were studied in detail. Based on the results from the above study, it can be inferred that Cd retards the pyrolysis velocity and deoxygenation behavior of the single biomass component as well as rice straw. Interestingly, Cd promotes the decomposition of the hemicellulose component of rice straw at a lower temperature, which provides important inspiration for the selective pyrolysis of the hemicellulose component in lignocellulosic biomass. In summary, the pyrolysis treatment of Cd-contaminated rice straw is more conducive to preparing biochar containing high oxygen content.

## Figures and Tables

**Table 1:** Proximate and ultimate analysis (wt.%) of three model components and rice straw.

Sample	Proximate Analysis (%)				Ultimate Analysis (%)					Cd (mg·kg^−1^)
	M ^1^	A ^2^	V ^3^	FC ^4^	C	H	O	H/C	O/C	
CE	3.88	0.02	91.43	4.67	42.4	6.3	51.2	1.79	0.90	-
XY	1.68	0.02	91.36	6.94	41.1	6.6	52.3	1.93	0.95	-
LG	12.26	15.59	47.14	25.01	47.5	4.6	28.3	1.15	0.45	-
RS	8.71	12.00	66.31	12.98	38.4	6.6	40.5	2.06	0.79	9.0

^1^ Moisture (air-dried basis). ^2^ Ash (air-dried basis) ^3^ Volatile matter (air-dried basis) ^4^ Fixed carbon (air-dried basis).

**Table 2:** C, H, and O content in pyrolysis residue of three model components.

Sample	C (%)	H (%)	O (%)
CE	42.44	6.32	51.20
RCE	89.55	2.26	7.40
RCE-5%Cd	88.67	2.04	8.64
XY	41.10	6.60	52.27
RXY	85.85	2.46	11.10
RXY-5%Cd	83.96	2.57	12.93
LG	47.51	4.55	43.87
RLG	60.95	1.98	33.25
RLG-5%Cd	60.91	1.84	33.82

Note: RCE, RXY, and RLG represent the pyrolysis residue of cellulose, xylan, and lignin, respectively. RCE-5%Cd, RXY-5%Cd, and RLG-5%Cd represent the pyrolysis residue of Cd-concentrated cellulose, xylan, and lignin, respectively.

## Data Availability

Not applicable.

## Supplementary Materials

The following supporting information can be downloaded at: https://www.mdpi.com/article/10.3390/ijerph19158953/s1, Figure S1: The pyrolysis TG/DTG curves of CdCl_2_·2.5H_2_O; Figure S2: Arrhenius plots of isoconversional method for biomass components samples. (a–f) are the CE and CE-Cd; (g–l) are the XY and XY-Cd; (m–r) are the LG and LG-Cd; Figure S3: Arrhenius plots of FWO method for Cd-contaminated rice straw. (a) RS, (b) RS-0.1%Cd, (c) RS-1%Cd, and (d) RS-5%Cd; Figure S4: Arrhenius plots of KAS method for Cd-contaminated rice straw. (a) RS, (b) RS-0.1%Cd, (c) RS-1%Cd, and (d) RS-5%Cd; Table S1: The content of cellulose, hemicellulose, and lignin in rice straw; Table S2: The measured content of Cd in the Cd-impregnated samples; Table S3: Fitting results based on the isoconversional methods; Table S4: The correlation between pyrolysis conversion (α) and pyrolysis temperature of cellulose, xylan, lignin, and rice straw; Table S5: Composition of pyrolytic volatiles from rice straw. Reference [46] is cited in Supplementary Materials.
