# Supplementary material for: Effect of Cd on Pyrolysis Velocity and Deoxygenation Characteristics of Rice Straw: Analogized with Cd-Impregnated Representative Biomass Components
Source: Int J Environ Res Public Health. 2022 Jul 23;19(15):8953. doi: 10.3390/ijerph19158953 (PMC9332023; doi:10.3390/ijerph19158953)
Supplement: Supplementary file 1 [file ijerph-19-08953-s001.zip › ijerph-1814399-SI.pdf]

# Effect of Cd on pyrolysis velocity and deoxygenation characteristics of rice straw: Analogized with Cd-impregnated representative biomass components

**SUPPLEMENTARY MATERIALS**

**Zhi Xu[1], Zhaohui Guo[1,*], Huimin Xie[1], Yulian Hu[1]**

[1] *Institute of Environmental Engineering, School of Metallurgy and Environment, Central South University, Changsha* 410083*, China*

**The determination of Cd content in Cd-impregnated samples**

The Cd-impregnated samples were digested by using $HNO_3$ and $HClO_4$ according to the methods reported by Zeng et al [1]. The digested solutions were analyzed by inductively coupled plasma-atomic emission spectrometry (ICP-AES, ICAP 7400, Thermo Fisher, USA). The measured content of Cd in Cd-impregnated samples is shown in Table S1.

References:

[1] P. Zeng, Z. Guo, X. Cao, et al. Phytostabilization potential of ornamental plants grown in soil contaminated with cadmium[J] Int. J. Phytoremediat., 2018, 20:311-320.

**Table S1.** The content of cellulose, hemicellulose, and lignin component in rice straw.

| Sample | Cellulose/% | Hemicellulose/% | Lignin/% |
|---|---|---|---|
| Rice straw | 39.7 | 24.8 | 18.5 |

**Table S2.** The measured content of Cd in Cd-impregnated samples.

| Samples | CE-5%Cd | XY-5%Cd | LG-5%Cd | RS-0.1%Cd | RS-1%Cd | RS-5%Cd |
|---|---|---|---|---|---|---|
| Content (%) | 4.69±0.22 | 4.92±0.35 | 4.76±0.29 | 0.11±0.03 | 1.17±0.24 | 4.96±0.31 |

**Table S3.** Fitting results based on the isoconversional methods.

| Sample | Method | | α | | | | | | | | | | | | |
|---|---|---|---|---|---|---|---|---|---|---|---|---|---|---|---|
| | | | 0.2 | 0.25 | 0.3 | 0.35 | 0.4 | 0.45 | 0.5 | 0.55 | 0.6 | 0.65 | 0.7 | 0.75 | 0.8 |
| CE | KAS | Intercept | 18.9 | 18.4 | 17.9 | 17.5 | 17.2 | 16.9 | 16.6 | 16.3 | 16.1 | 15.9 | 15.6 | 15.5 | 15.2 |
| | | Slope | -17.6 | -17.4 | -17.2 | -17.0 | -16.9 | -16.8 | -16.7 | -16.5 | -16.4 | -16.4 | -16.3 | -16.2 | -16.1 |
| | | $R^2$ | 0.9991 | 0.9988 | 0.9986 | 0.9983 | 0.9982 | 0.9981 | 0.9979 | 0.9977 | 0.9975 | 0.9975 | 0.9973 | 0.9973 | 0.9971 |
| | FWO | Intercept | 33.7 | 33.2 | 32.8 | 32.4 | 32.1 | 31.7 | 31.5 | 31.2 | 31.0 | 30.8 | 30.5 | 30.4 | 30.1 |
| | | Slope | -18.9 | -18.7 | -18.5 | -18.3 | -18.2 | -18.0 | -17.9 | -17.8 | -17.7 | -17.6 | -17.5 | -17.5 | -17.4 |
| | | $R^2$ | 0.9993 | 0.9990 | 0.9989 | 0.9986 | 0.9985 | 0.9984 | 0.9982 | 0.9981 | 0.9979 | 0.9979 | 0.9978 | 0.9978 | 0.9976 |
| | Friedman | Intercept | 25.2 | 24.9 | 24.7 | 24.8 | 23.9 | 23.8 | 23.5 | 24.0 | 23.8 | 23.6 | 23.5 | 23.6 | 23.4 |
| | | Slope | -16.2 | -16.0 | -15.9 | -16.0 | -15.4 | -15.3 | -15.2 | -15.6 | -15.5 | -15.4 | -15.4 | -15.5 | -15.6 |
| | | $R^2$ | 0.9948 | 0.9940 | 0.9965 | 0.9932 | 0.9962 | 0.9960 | 0.9952 | 0.9934 | 0.9944 | 0.9941 | 0.9961 | 0.9949 | 0.9961 |
| CE-5%Cd | KAS | Intercept | 45.1 | 44.3 | 48.5 | 46.7 | 44.9 | 43.4 | 41.6 | 40.0 | 38.1 | 36.2 | 33.9 | 31.2 | 27.6 |
| | | Slope | -32.2 | -32.0 | -34.8 | -33.9 | -33.1 | -32.3 | -31.4 | -30.6 | -29.6 | -28.6 | -27.3 | -25.8 | -23.8 |
| | | $R^2$ | 0.6639 | 0.7117 | 0.9537 | 0.9652 | 0.9740 | 0.9819 | 0.9887 | 0.9948 | 0.9988 | 0.9999 | 0.9952 | 0.9793 | 0.9408 |
| | FWO | Intercept | 59.9 | 59.1 | 63.3 | 61.5 | 59.8 | 58.2 | 56.4 | 54.8 | 52.9 | 51.1 | 488 | 46.0 | 42.5 |
| | | Slope | -33.4 | -33.2 | -36.0 | -35.1 | -34.3 | -33.5 | -32.6 | -31.8 | -30.8 | -29.8 | -28.5 | -27.0 | -25.0 |
| | | $R^2$ | 0.6823 | 0.7284 | 0.9567 | 0.9676 | 0.9758 | 0.9832 | 0.9896 | 0.9952 | 0.9989 | 0.9999 | 0.9956 | 0.9811 | 0.9463 |
| | Friedman | Intercept | 50.9 | 49.1 | 51.6 | 50.8 | 48.4 | 45.8 | 44.6 | 42.7 | 40.2 | 38.2 | 35.0 | 32.9 | 30.4 |
| | | Slope | -30.8 | -29.9 | -31.6 | -31.3 | -30.0 | -28.5 | -27.9 | -26.9 | -25.5 | -24.4 | -22.6 | -21.6 | -20.3 |
| | | $R^2$ | 0.7427 | 0.8066 | 0.9874 | 0.9932 | 0.9979 | 0.9986 | 0.9987 | 0.9949 | 0.9869 | 0.9764 | 0.9547 | 0.9190 | 0.8753 |
| XY | KAS | Intercept | 19.2 | 19.7 | 20.6 | 21.9 | 22.8 | 25.3 | 26.5 | 27.7 | 29.2 | 30.7 | 35.2 | 57.3 | 230.0 |
| | | Slope | -15.4 | -15.8 | -16.6 | -17.5 | -18.3 | -19.9 | -20.7 | -21.6 | -22.6 | -23.7 | -26.6 | -40.2 | -147.3 |
| | | $R^2$ | 0.9985 | 0.9979 | 0.9972 | 0.9970 | 0.9982 | 0.9997 | 0.9983 | 0.9962 | 0.9926 | 0.9921 | 0.9924 | 0.9448 | 0.9999 |
| | FWO | Intercept | 33.7 | 34.2 | 35.2 | 36.5 | 37.5 | 39.9 | 41.2 | 42.4 | 43.9 | 45.5 | 50.0 | 72.0 | 244.8 |
| | | Slope | -16.4 | -16.9 | -17.7 | -18.6 | -19.4 | -21.0 | -21.9 | -22.7 | -23.8 | -24.9 | -27.8 | -41.4 | -148.6 |

| | | | | | | | | | | | | | | | |
|---|---|---|---|---|---|---|---|---|---|---|---|---|---|---|---|
| | | $R^2$ | 0.9989 | 0.9982 | 0.9976 | 0.9974 | 0.9984 | 0.9997 | 0.9984 | 0.9965 | 0.9933 | 0.9928 | 0.9930 | 0.9479 | 0.9999 |
| | Friedman | Intercept | 28.0 | 27.3 | 33.0 | 32.5 | 36.7 | 40.9 | 40.4 | 40.7 | 42.8 | 46.1 | 59.9 | 96.9 | 300.9 |
| | | Slope | -16.0 | -15.8 | -19.3 | -19.2 | -21.7 | -24.1 | -24.0 | -24.3 | -25.7 | -27.9 | -36.4 | -59.4 | -186.7 |
| | | $R^2$ | 0.9666 | 0.9766 | 0.9406 | 0.9956 | 0.9975 | 0.9989 | 0.9893 | 0.9831 | 0.9813 | 0.9888 | 0.9117 | 0.9259 | 0.9733 |
| XY-5%Cd | KAS | Intercept | 40.4 | 47.2 | 49.5 | 56.0 | 61.0 | 68.1 | 79.8 | 145.1 | 231.5 | 389.8 | 186.8 | 167.0 | 119.0 |
| | | Slope | -25.8 | -29.7 | -31.3 | -35.0 | -38.1 | -42.2 | -49.1 | -86.0 | -137.7 | -235.3 | -119.9 | -111.4 | -84.6 |
| | | $R^2$ | 0.8829 | 0.9230 | 0.9063 | 0.8586 | 0.8374 | 0.7951 | 0.8309 | 0.7671 | 0.8504 | 0.9569 | 0.8898 | 0.8773 | 0.7299 |
| | FWO | Intercept | 54.9 | 61.8 | 64.1 | 70.5 | 75.6 | 82.7 | 94.4 | 159.8 | 246.2 | 404.6 | 201.6 | 181.9 | 133.9 |
| | | Slope | -26.8 | -30.8 | -32.3 | -36.1 | -39.1 | -43.3 | -50.2 | -87.1 | -138.8 | -236.5 | -121.2 | -112.7 | -85.9 |
| | | $R^2$ | 0.8910 | 0.9279 | 0.9120 | 0.8661 | 0.8453 | 0.8039 | 0.8374 | 0.7721 | 0.8526 | 0.9573 | 0.8918 | 0.8798 | 0.7366 |
| | Friedman | Intercept | 69.0 | 66.8 | 58.2 | 63.2 | 75.6 | 82.6 | 116.0 | 227.2 | 351.2 | 455.1 | 166.5 | 145.5 | 139.4 |
| | | Slope | -36.6 | -36.0 | -31.8 | -34.6 | -41.5 | -45.7 | -64.6 | -127.3 | -201.9 | -269.7 | -103.5 | -93.6 | -93.6 |
| | | $R^2$ | 0.9453 | 0.9373 | 0.8133 | 0.8303 | 0.8244 | 0.8019 | 0.8595 | 0.8267 | 0.7699 | 0.8742 | 0.8114 | 0.8063 | 0.7086 |
| LG | KAS | Intercept | 55.3 | 53.3 | 52.8 | 50.8 | 48.3 | 47.0 | 45.9 | 45.7 | 46.9 | 49.6 | 56.4 | 72.4 | 51.1 |
| | | Slope | -35.8 | -36.0 | -36.7 | -36.3 | -35.5 | -35.3 | -35.2 | -35.7 | -37.1 | -39.7 | -45.2 | -57.7 | -44.7 |
| | | $R^2$ | 0.9912 | 0.9943 | 0.9966 | 0.9999 | 0.9996 | 0.9989 | 0.9981 | 0.9974 | 0.9975 | 0.9960 | 0.9928 | 0.9318 | 0.6745 |
| | FWO | Intercept | 69.9 | 68.0 | 67.6 | 65.6 | 63.2 | 61.9 | 60.8 | 60.6 | 61.8 | 64.6 | 71.5 | 87.5 | 66.3 |
| | | Slope | -36.9 | -37.1 | -37.9 | -37.5 | -36.7 | -36.5 | -36.4 | -37.0 | -38.4 | -41.0 | -46.5 | -59.1 | -46.2 |
| | | $R^2$ | 0.9917 | 0.9946 | 0.9968 | 0.9999 | 0.9996 | 0.9990 | 0.9982 | 0.9976 | 0.9976 | 0.9963 | 0.9932 | 0.9349 | 0.6907 |
| | Friedman | Intercept | 63.8 | 67.5 | 60.2 | 58.2 | 52.8 | 53.3 | 51.6 | 61.0 | 52.4 | 55.7 | 77.7 | 89.3 | 70.9 |
| | | Slope | -36.9 | -40.1 | -36.9 | -36.3 | -33.6 | -34.6 | -34.0 | -40.7 | -35.8 | -38.7 | -54.6 | -64.6 | -54.3 |
| | | $R^2$ | 0.9020 | 0.9943 | 0.9922 | 0.9967 | 0.9873 | 0.9957 | 0.9995 | 0.9709 | 0.9866 | 0.9785 | 0.9442 | 0.8405 | 0.4977 |
| LG-5%Cd | KAS | Intercept | 51.1 | 57.3 | 60.7 | 57.8 | 51.6 | 47.6 | 47.6 | 51.0 | 64.3 | 89.6 | 77.7 | 77.2 | 62.2 |
| | | Slope | -33.5 | -38.3 | -41.5 | -40.9 | -38.0 | -36.4 | -37.1 | -40.2 | -50.2 | -69.4 | -63.5 | -65.3 | -56.0 |
| | | $R^2$ | 0.8608 | 0.9075 | 0.9630 | 0.9861 | 0.9940 | 0.9961 | 0.9961 | 0.9921 | 0.9879 | 0.9734 | 0.9559 | 0.9916 | 0.9787 |
| | FWO | Intercept | 65.7 | 72.0 | 75.4 | 72.6 | 66.4 | 62.5 | 62.6 | 66.0 | 79.4 | 104.6 | 92.9 | 92.4 | 77.5 |

| | | | | | | | | | | | | | | | |
|---|---|---|---|---|---|---|---|---|---|---|---|---|---|---|---|
| | | Slope | -34.8 | -39.4 | -42.7 | -42.1 | -39.3 | -37.6 | -38.4 | -41.5 | -51.6 | -70.8 | -64.9 | -66.8 | -57.5 |
| | | $R^2$ | 0.8688 | 0.9126 | 0.9650 | 0.9869 | 0.9944 | 0.9964 | 0.9964 | 0.9927 | 0.9885 | 0.9744 | 0.9578 | 0.9920 | 0.9798 |
| | Friedman | Intercept | 51.3 | 64.0 | 68.3 | 69.4 | 54.2 | 52.7 | 58.1 | 62.1 | 81.0 | 133.6 | 80.5 | 57.1 | 64.0 |
| | | Slope | -30.0 | -38.2 | -41.9 | -43.6 | -35.2 | -34.9 | -39.1 | -42.6 | -56.6 | -95.1 | -60.4 | -45.2 | -52.0 |
| | | $R^2$ | 0.7636 | 0.9402 | 0.9168 | 0.9993 | 0.9932 | 0.9951 | 0.9925 | 0.9740 | 0.9879 | 0.999 | 0.9778 | 0.9169 | 0.9155 |
| RS | KAS | Intercept | 22.1 | 23.8 | 23.4 | 23.8 | 25.9 | 25.4 | 24.7 | 25.2 | 25.6 | 24.2 | 24.8 | 26.6 | 26.0 |
| | | Slope | -17.7 | -19.0 | -19.0 | -19.5 | -20.9 | -20.8 | -20.6 | -21.1 | -21.5 | -20.8 | -21.3 | -22.7 | -22.7 |
| | | $R^2$ | 0.9892 | 0.9930 | 0.9971 | 0.9957 | 0.9886 | 0.9909 | 0.9941 | 0.9911 | 0.9891 | 0.9955 | 0.9911 | 0.9772 | 0.9413 |
| | FWO | Intercept | 36.8 | 38.5 | 38.1 | 38.6 | 40.6 | 40.1 | 39.5 | 40.0 | 40.4 | 39.0 | 39.6 | 41.4 | 40.8 |
| | | Slope | -18.8 | -20.1 | -20.2 | -20.7 | -22.1 | -22.0 | -21.8 | -22.3 | -22.7 | -22.1 | -22.6 | -23.0 | -23.9 |
| | | $R^2$ | 0.9903 | 0.9937 | 0.9974 | 0.9961 | 0.9896 | 0.9917 | 0.9947 | 0.9920 | 0.9901 | 0.9959 | 0.9919 | 0.9793 | 0.9468 |
| RS-0.1%Cd | KAS | Intercept | 25.4 | 24.2 | 26.1 | 26.9 | 27.5 | 27.4 | 26.4 | 26.8 | 27.5 | 28.4 | 27.7 | 31.1 | 25.8 |
| | | Slope | -19.6 | -19.2 | -20.6 | -21.3 | -21.9 | -22.1 | -21.7 | -22.1 | -22.7 | -23.4 | -23.2 | -25.6 | -22.6 |
| | | $R^2$ | 0.9987 | 0.9978 | 0.9993 | 0.9998 | 0.9993 | 0.9976 | 0.9975 | 0.9918 | 0.9889 | 0.9889 | 0.9937 | 0.9724 | 0.9755 |
| | FWO | Intercept | 40.1 | 38.8 | 40.8 | 41.6 | 42.3 | 42.1 | 41.2 | 41.6 | 42.3 | 43.2 | 42.6 | 46.0 | 40.7 |
| | | Slope | -20.7 | -20.3 | -21.7 | -22.5 | -23.1 | -23.3 | -22.2 | -23.3 | -23.9 | -24.7 | -24.5 | -26.8 | -23.8 |
| | | $R^2$ | 0.9988 | 0.9981 | 0.9994 | 0.9998 | 0.9994 | 0.9978 | 0.9977 | 0.9926 | 0.9899 | 0.9899 | 0.9943 | 0.9749 | 0.9778 |
| RS-1%Cd | KAS | Intercept | 38.6 | 38.8 | 37.9 | 36.9 | 38.3 | 38.1 | 35.5 | 32.8 | 32.5 | 32.1 | 32.3 | 33.3 | 55.8 |
| | | Slope | -26.8 | -27.3 | -27.3 | -27.1 | -28.3 | -28.4 | -27.2 | -25.8 | -25.8 | -25.7 | -26.0 | -26.9 | -41.8 |
| | | $R^2$ | 0.9769 | 0.9868 | 0.9881 | 0.9835 | 0.9962 | 0.9998 | 0.9994 | 0.9995 | 0.9999 | 0.9995 | 0.9995 | 0.9963 | 0.9072 |
| | FWO | Intercept | 53.2 | 53.5 | 52.6 | 51.6 | 53.1 | 52.8 | 50.3 | 47.6 | 47.4 | 46.9 | 47.1 | 48.2 | 70.7 |
| | | Slope | -27.9 | -28.5 | -28.4 | -28.2 | -29.4 | -29.6 | -28.4 | -27.0 | -27.0 | -26.9 | -27.3 | -28.2 | -43.0 |
| | | $R^2$ | 0.9787 | 0.9879 | 0.9891 | 0.9849 | 0.9965 | 0.9998 | 0.9994 | 0.9996 | 0.9999 | 0.9993 | 0.9995 | 0.9967 | 0.9124 |
| RS-5%Cd | KAS | Intercept | 34.9 | 36.0 | 37.8 | 36.5 | 36.8 | 35.0 | 34.0 | 33.2 | 34.4 | 36.8 | 38.2 | 51.5 | 72.0 |
| | | Slope | -24.6 | -25.5 | -26.9 | -26.6 | -27.2 | -26.5 | -26.3 | -25.9 | -26.8 | -28.5 | -29.5 | -38.3 | -53.3 |
| | | $R^2$ | 0.9657 | 0.9561 | 0.9618 | 0.9770 | 0.9682 | 0.9611 | 0.9594 | 0.9772 | 0.9788 | 0.9844 | 0.9700 | 0.9937 | 0.9825 |

| | | | | | | | | | | | | | | |
|---|---|---|---|---|---|---|---|---|---|---|---|---|---|---|
| FWO | Intercept | 49.5 | 50.6 | 52.5 | 51.2 | 51.6 | 49.7 | 49.1 | 48.0 | 49.3 | 51.6 | 53.0 | 66.3 | 86.9 |
| | Slope | -25.7 | -26.6 | -28.1 | -27.8 | -28.4 | -27.6 | -27.5 | -27.1 | -28.0 | -29.7 | -30.8 | -39.5 | -54.6 |
| | $R^2$ | 0.9684 | 0.9594 | 0.9647 | 0.9788 | 0.9706 | 0.9642 | 0.9626 | 0.9791 | 0.9801 | 0.9855 | 0.9722 | 0.9941 | 0.9833 |

**Table S4.** The correlation between pyrolysis conversion (α) and pyrolysis temperature of cellulose, xylan, lignin, and rice straw.

| Pyrolysis conversion (α) | | 0.2 | 0.25 | 0.3 | 0.35 | 0.4 | 0.45 | 0.5 | 0.55 | 0.6 | 0.65 | 0.7 | 0.75 | 0.8 |
|---|---|---|---|---|---|---|---|---|---|---|---|---|---|---|
| Sample | β/°C·min$^{-1}$ | Pyrolysis temperature/°C | | | | | | | | | | | | |
| CE | 5 | 314.8 | 317.5 | 319.8 | 321.8 | 323.6 | 325.2 | 326.8 | 328.4 | 330.0 | 331.5 | 333.2 | 335.0 | 337.0 |
| | 10 | 327.5 | 330.4 | 332.9 | 335.0 | 337.0 | 338.8 | 340.6 | 342.3 | 344.0 | 345.8 | 347.6 | 349.6 | 351.7 |
| | 20 | 340.4 | 343.6 | 346.3 | 348.6 | 350.7 | 352.7 | 354.5 | 356.4 | 358.2 | 360.1 | 362.0 | 364.1 | 366.3 |
| | 50 | 360.3 | 364.0 | 367.2 | 370.1 | 372.5 | 374.9 | 377.1 | 379.3 | 381.5 | 383.6 | 385.9 | 388.2 | 390.8 |
| CE-5%Cd | 5 | 304.7 | 308.5 | 311.7 | 314.7 | 317.3 | 319.8 | 322.2 | 324.6 | 326.9 | 329.3 | 331.9 | 334.7 | 338.3 |
| | 10 | 308.9 | 313.1 | 316.9 | 320.4 | 323.6 | 326.6 | 329.5 | 332.3 | 335.1 | 337.9 | 340.9 | 344.1 | 347.9 |
| | 20 | 307.9 | 312.7 | 321.6 | 325.4 | 328.9 | 332.4 | 335.8 | 339.3 | 342.9 | 346.9 | 351.5 | 356.9 | 364.1 |
| | 50 | 324.9 | 329.8 | 334.2 | 338.0 | 341.7 | 345.1 | 348.5 | 351.8 | 355.2 | 358.7 | 362.5 | 366.8 | 371.9 |
| XY | 5 | 238.2 | 245.6 | 253.8 | 261.8 | 268.7 | 274.5 | 279.6 | 284.2 | 288.8 | 293.8 | 300.9 | 316.0 | 337.8 |
| | 10 | 249.0 | 256.6 | 264.4 | 272.1 | 278.6 | 284.3 | 289.3 | 294.1 | 298.5 | 303.0 | 308.5 | 318.1 | 339.5 |
| | 20 | 260.3 | 267.5 | 275.1 | 282.6 | 289.5 | 295.3 | 300.5 | 305.1 | 309.5 | 314.0 | 318.9 | 325.6 | 341.3 |
| | 50 | 277.8 | 285.2 | 292.6 | 299.7 | 305.8 | 309.3 | 313.5 | 317.3 | 320.7 | 324.7 | 328.9 | 334.5 | 343.6 |
| XY-5%Cd | 5 | 232.2 | 240.4 | 246.2 | 251.6 | 257.0 | 262.3 | 268.8 | 278.4 | 294.7 | 313.9 | 332.4 | 351.4 | 374.9 |
| | 10 | 233.2 | 242.1 | 247.9 | 253.3 | 258.6 | 264.0 | 270.2 | 278.7 | 295.7 | 314.4 | 335.2 | 355.4 | 381.7 |
| | 20 | 244.8 | 251.7 | 257.9 | 263.4 | 268.3 | 273.2 | 278.2 | 283.7 | 298.7 | 315.9 | 337.8 | 357.4 | 383.4 |
| | 50 | 251.5 | 258.6 | 263.4 | 266.6 | 270.7 | 274.4 | 279.8 | 284.5 | 299.1 | 317.0 | 338.8 | 359.0 | 385.0 |
| LG | 5 | 267.6 | 286.1 | 301.2 | 312.6 | 323.0 | 332.8 | 342.7 | 353.3 | 364.7 | 377.5 | 393.5 | 416.5 | 447.4 |
| | 10 | 271.9 | 291.2 | 306.4 | 319.0 | 329.9 | 339.9 | 349.9 | 360.5 | 371.9 | 384.2 | 398.6 | 418.4 | 447.3 |
| | 20 | 277.8 | 297.1 | 312.9 | 325.6 | 337.1 | 347.6 | 358.1 | 369.0 | 380.4 | 392.7 | 406.6 | 424.7 | 451.1 |
| | 50 | 286.2 | 306.1 | 321.8 | 334.4 | 346.1 | 356.8 | 367.4 | 378.5 | 389.8 | 401.7 | 415.7 | 434.1 | 468.6 |
| LG-5%Cd | 5 | 269.4 | 288.7 | 305.7 | 320.5 | 333.2 | 345.3 | 357.8 | 372.3 | 390.6 | 414.2 | 439.0 | 462.1 | 485.9 |
| | 10 | 271.3 | 291.7 | 309.8 | 325.6 | 339.6 | 352.2 | 364.8 | 378.9 | 396.3 | 419.0 | 444.6 | 469.0 | 492.8 |
| | 20 | 275.6 | 295.6 | 314.1 | 330.6 | 345.3 | 358.7 | 371.5 | 385.0 | 401.1 | 422.0 | 447.4 | 473.4 | 497.7 |
| | 50 | 287.8 | 306.8 | 324.1 | 340.4 | 355.7 | 369.7 | 382.7 | 396.4 | 411.0 | 430.1 | 457.6 | 481.5 | 509.8 |
| RS | 5 | 261.9 | 271.5 | 279.0 | 285.5 | 291.5 | 297.0 | 302.0 | 306.6 | 310.8 | 315.3 | 319.9 | 325.8 | 335.1 |
| | 10 | 274.9 | 283.7 | 290.8 | 297.5 | 303.7 | 309.1 | 314.5 | 319.1 | 323.7 | 327.9 | 332.9 | 339.5 | 352.0 |
| | 20 | 286.0 | 294.5 | 302.0 | 308.7 | 314.5 | 320.3 | 325.3 | 330.3 | 334.5 | 339.5 | 344.5 | 350.3 | 361.0 |
| | 50 | 299.3 | 307.8 | 316.2 | 322.6 | 326.8 | 333.0 | 339.3 | 343.5 | 347.6 | 353.9 | 358.1 | 362.3 | 372.6 |
| RS-0.1%Cd | 5 | 264.6 | 273.6 | 281.3 | 288.2 | 294.7 | 300.5 | 305.7 | 310.5 | 315.1 | 319.9 | 325.1 | 332.8 | 335.1 |
| | 10 | 275.1 | 283.5 | 291.4 | 298.0 | 304.7 | 310.9 | 315.9 | 319.7 | 324.7 | 328.9 | 333.5 | 338.5 | 348.4 |
| | 20 | 284.4 | 293.6 | 301.1 | 308.6 | 315.3 | 321.9 | 327.7 | 332.7 | 337.7 | 341.9 | 346.1 | 351.9 | 361.1 |
| | 50 | 299.1 | 309.7 | 316.1 | 322.4 | 328.6 | 334.9 | 341.1 | 345.3 | 349.4 | 353.6 | 359.9 | 364.1 | 372.4 |
| RS-1%Cd | 5 | 270.0 | 277.9 | 285.7 | 293.2 | 299.6 | 305.2 | 309.8 | 314.0 | 317.8 | 321.5 | 325.7 | 332.4 | 352.1 |
| | 10 | 274.6 | 283.4 | 291.3 | 298.7 | 306.2 | 312.9 | 317.9 | 322.5 | 326.7 | 330.4 | 334.6 | 340.4 | 354.1 |
| | 20 | 283.0 | 291.4 | 299.8 | 307.2 | 314.7 | 321.3 | 327.1 | 332.1 | 336.3 | 340.5 | 344.7 | 349.7 | 360.4 |
| | 50 | 294.6 | 303.1 | 311.5 | 319.9 | 326.2 | 332.4 | 338.6 | 344.8 | 349.0 | 353.2 | 357.4 | 363.7 | 372.0 |
| RS-5%Cd | 5 | 261.3 | 269.5 | 277.7 | 285.8 | 293.6 | 299.9 | 305.2 | 309.6 | 314.2 | 319.5 | 324.2 | 337.4 | 365.9 |
| | 10 | 271.1 | 279.1 | 287.0 | 295.3 | 303.2 | 310.7 | 316.2 | 320.8 | 325.0 | 329.1 | 334.6 | 344.5 | 372.9 |
| | 20 | 280.2 | 288.6 | 296.1 | 304.4 | 312.7 | 320.2 | 326.0 | 330.2 | 334.4 | 338.6 | 343.6 | 351.9 | 376.8 |
| | 50 | 287.6 | 295.2 | 302.9 | 312.5 | 320.2 | 328.0 | 333.8 | 339.7 | 343.7 | 347.7 | 351.7 | 359.7 | 383.9 |

**Table S5.** Composition of pyrolytic volatiles from rice straw.

| Sample | RS | |
|---|---|---|
| Number | Components | Relative content/% |
| 1 | Alanine | 38.37 |
| 2 | Acetone | 23.16 |
| 3 | 3-Pentanone | 9.04 |
| 4 | Acetic acid | 8.43 |
| 5 | Cyclopropyl carbinol | 5.56 |
| 6 | Methyl formate | 4.18 |
| 7 | Cyclohexanone | 2.52 |
| 8 | 4-Hydroxy-3-methylacetophenone | 2.41 |
| 9 | n-Hexadecanoic acid | 1.17 |
| 10 | 2-Propanone, 1-hydroxy- | 0.82 |
| 11 | 9-Octadecenamide, (Z)- | 0.46 |
| 12 | Oleic Acid | 0.38 |
| 13 | 9-Octadecen-1-ol, (Z)- | 0.37 |
| 14 | Phenol, 2-methoxy- | 0.33 |
| 15 | Dibutyl phthalate | 0.28 |
| 16 | Heptane, 3-methylene- | 0.27 |
| 17 | Cyclohexane, 2-butyl-1,1,3-trimethyl- | 0.27 |
| 18 | Vanillin | 0.26 |
| 19 | 3',5'-Dimethoxyacetophenone | 0.24 |
| 20 | 2-Pentadecanone, 6,10,14-trimethyl- | 0.23 |
| 21 | Dibutyl phthalate | 0.22 |
| 22 | Ethanone, 1-(4-hydroxy-3,5-dimethoxyphenyl)- | 0.21 |
| 23 | Docosanoic acid | 0.21 |
| 24 | .beta.-D-Glucopyranose, 1,6-anhydro- | 0.17 |
| 25 | 4,8,12,16-Tetramethylheptadecan-4-olide | 0.1 |
| 26 | Cyclododecanol | 0.08 |
| 27 | Cyclohexane, undecyl- | 0.08 |
| 28 | Pentadecanal- | 0.06 |
| 29 | Furan, 2-hexyl- | 0.06 |
| 30 | 9-Tricosene, (Z)- | 0.06 |

| Sample | RS-0.1%Cd | |
|---|---|---|
| Number | Components | Relative amount/% |
| 1 | Alanine | 52.89 |
| 2 | Acetone | 19.37 |
| 3 | Cyclopropyl carbinol | 9.18 |
| 4 | 4-Hydroxy-3-methylacetophenone | 3.8 |
| 5 | Benzofuran, 2,3-dihydro- | 2.38 |
| 6 | Methyl formate | 2.28 |
| 7 | 3-Pentanone | 1.77 |
| 8 | n-Hexadecanoic acid | 1.6 |
| 9 | Phenol, 2-methoxy- | 0.9 |
| 10 | Phenol, 2,6-dimethoxy- | 0.68 |
| 11 | 2(5H)-Furanone | 0.54 |

| | | |
|---|---|---|
| 12 | 9-Octadecen-1-ol, (Z)- | 0.47 |
| 13 | Oleic Acid | 0.47 |
| 14 | 1-Dodecanol, 3,7,11-trimethyl- | 0.41 |
| 15 | 1,2-Benzenedicarboxylic acid, diundecyl ester | 0.39 |
| 16 | 3',5'-Dimethoxyacetophenone | 0.37 |
| 17 | 2-Cyclopenten-1-one, 3-ethyl-2-hydroxy- | 0.27 |
| 18 | .beta.-D-Glucopyranose, 1,6-anhydro- | 0.27 |
| 19 | 2-Pentadecanone, 6,10,14-trimethyl- | 0.27 |
| 20 | Ethanone, 1-(4-hydroxy-3,5-dimethoxyphenyl)- | 0.25 |
| 21 | 4,8,12,16-Tetramethylheptadecan-4-olide | 0.21 |
| 22 | Tetracontane | 0.21 |
| 23 | 13-Docosenamide, (Z)- | 0.2 |
| 24 | 2-Nonenal, (E)- | 0.16 |
| 25 | Cyclohexane, undecyl- | 0.16 |
| 26 | Dibutyl phthalate | 0.14 |
| 27 | 4,8,12,16-Tetramethylheptadecan-4-olide | 0.12 |
| 28 | 9-Tricosene, (Z)- | 0.1 |
| 29 | 9-Octadecenamide, (Z)- | 0.07 |
| 30 | Tetracosane | 0.07 |

| Sample | RS-1%Cd | |
|---|---|---|
| Number | Components | Relative amount/% |
| 1 | Alanine | 43.8 |
| 2 | Acetone | 15.61 |
| 3 | Cyclopropyl carbinol | 8.03 |
| 4 | Cyclohexanone | 4.42 |
| 5 | 4-Hydroxy-3-methylacetophenone | 4.07 |
| 6 | Methyl formate | 2.71 |
| 7 | Benzofuran, 2,3-dihydro- | 2.67 |
| 8 | N,N-Diamylmethylamine | 2.24 |
| 9 | 3-Pentanone | 1.85 |
| 10 | Phenol, 2,6-dimethoxy- | 1.72 |
| 11 | .beta.-D-Glucopyranose, 1,6-anhydro- | 1.62 |
| 12 | n-Hexadecanoic acid | 1.54 |
| 13 | 2(5H)-Furanone | 1.28 |
| 14 | 1,3-Propanediol, 2-ethyl-2-(hydroxymethyl)- | 1.09 |
| 15 | 2-Propanone, 1-hydroxy- | 0.97 |
| 16 | Phenol, 2-methoxy- | 0.88 |
| 17 | Acetic acid | 0.86 |
| 18 | Methacrylic acid, ethyl ester | 0.78 |
| 19 | 2-Methoxy-6-methylaniline | 0.69 |
| 20 | Oleic Acid | 0.47 |
| 21 | Pentadecanal- | 0.41 |
| 22 | 3',5'-Dimethoxyacetophenone | 0.33 |
| 23 | 4-Heptanol, 2-methyl- | 0.31 |
| 24 | 3',5'-Dihydroxyacetophenone | 0.27 |
| 25 | Phenol, 2,6-dimethoxy-4-(2-propenyl)- | 0.25 |

| | | |
|---|---|---|
| 26 | 2-Cyclopenten-1-one, 3-ethyl-2-hydroxy- | 0.24 |
| 27 | Ethanone, 1-(4-hydroxy-3,5-dimethoxyphenyl)- | 0.24 |
| 28 | Heptanal | 0.22 |
| 29 | Phenol, 4-ethyl-2-methoxy- | 0.22 |
| 30 | Citronellyl isobutyrate | 0.21 |
| Sample | RS-5%Cd | |
| Number | Components | Relative amount/% |
| 1 | Alanine | 34.76 |
| 2 | .beta.-D-Glucopyranose, 1,6-anhydro- | 15.27 |
| 3 | Acetone | 13.83 |
| 4 | N,N-Diamylmethylamine | 7.05 |
| 5 | 4-Hydroxy-3-methylacetophenone | 3.36 |
| 6 | Heptanal | 3.29 |
| 7 | Methyl formate | 2.68 |
| 8 | D-erythro-Pentose, 2-deoxy- | 2.31 |
| 9 | Benzofuran, 2,3-dihydro- | 2.3 |
| 10 | Furfural | 1.94 |
| 11 | 1,3-Propanediol, 2-(hydroxymethyl)-2-nitro- | 1.71 |
| 12 | Acetic acid | 0.95 |
| 13 | Cyclopropyl carbinol | 0.87 |
| 14 | Ethyl cyclopropanecarboxylate | 0.84 |
| 15 | 3-Pentanone | 0.83 |
| 16 | n-Hexadecanoic acid | 0.83 |
| 17 | Pentanedioic acid | 0.76 |
| 18 | Phloroglucitol | 0.75 |
| 19 | 1,2-Cyclopentanediol, 3-methyl- | 0.72 |
| 20 | Oxirane, 2,2'-[1,4-butanediylbis(oxymethylene)]bis- | 0.69 |
| 21 | 1,2-Cyclopentanedione, 3-methyl- | 0.61 |
| 22 | 3-Heptanol | 0.53 |
| 23 | Phenol, 2-methoxy- | 0.46 |
| 24 | 2-Propanone, 1-hydroxy- | 0.45 |
| 25 | 5-Hydroxymethylfurfural | 0.39 |
| 26 | Phenol, 4-ethyl-2-methoxy- | 0.39 |
| 27 | 1,4-Cyclohexanedione | 0.38 |
| 28 | Vanillin | 0.38 |
| 29 | Furaneol | 0.34 |
| 30 | Maltol | 0.33 |

*Note*: The composition of volatiles was analyzed using PY-GC/MS (EGA/PY-3030D, Frontier, Japan; TRACE 1310/TRACE ISQ, Thermo Scientific, USA).

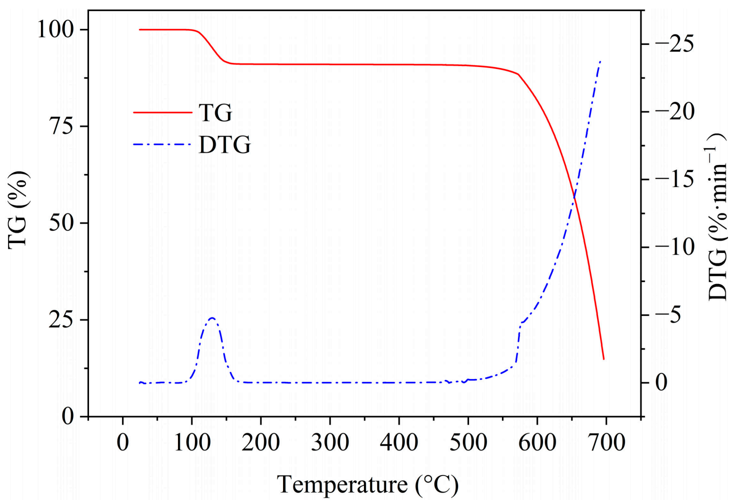


**Figure S1**. The pyrolysis TG/DTG curves of $CdCl_2 \cdot 2.5H_2O$.

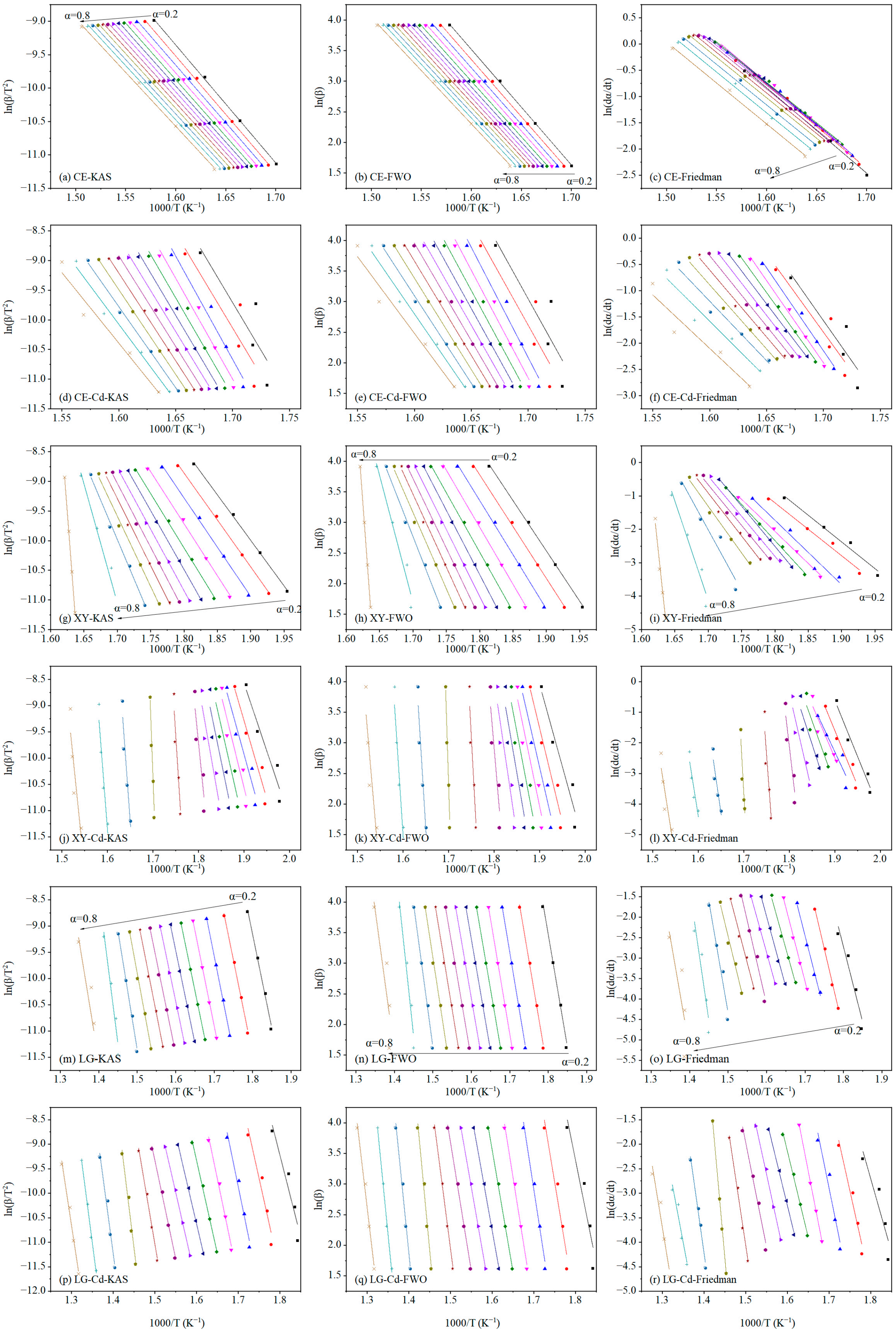


**Figure S2.** Arrhenius plots of isoconversional method for biomass components samples. (a)-(f) are the CE and CE-5%Cd; (g)-(l) are the XY and XY-5%Cd; (m)-(r) are the LG and LG-5%Cd.

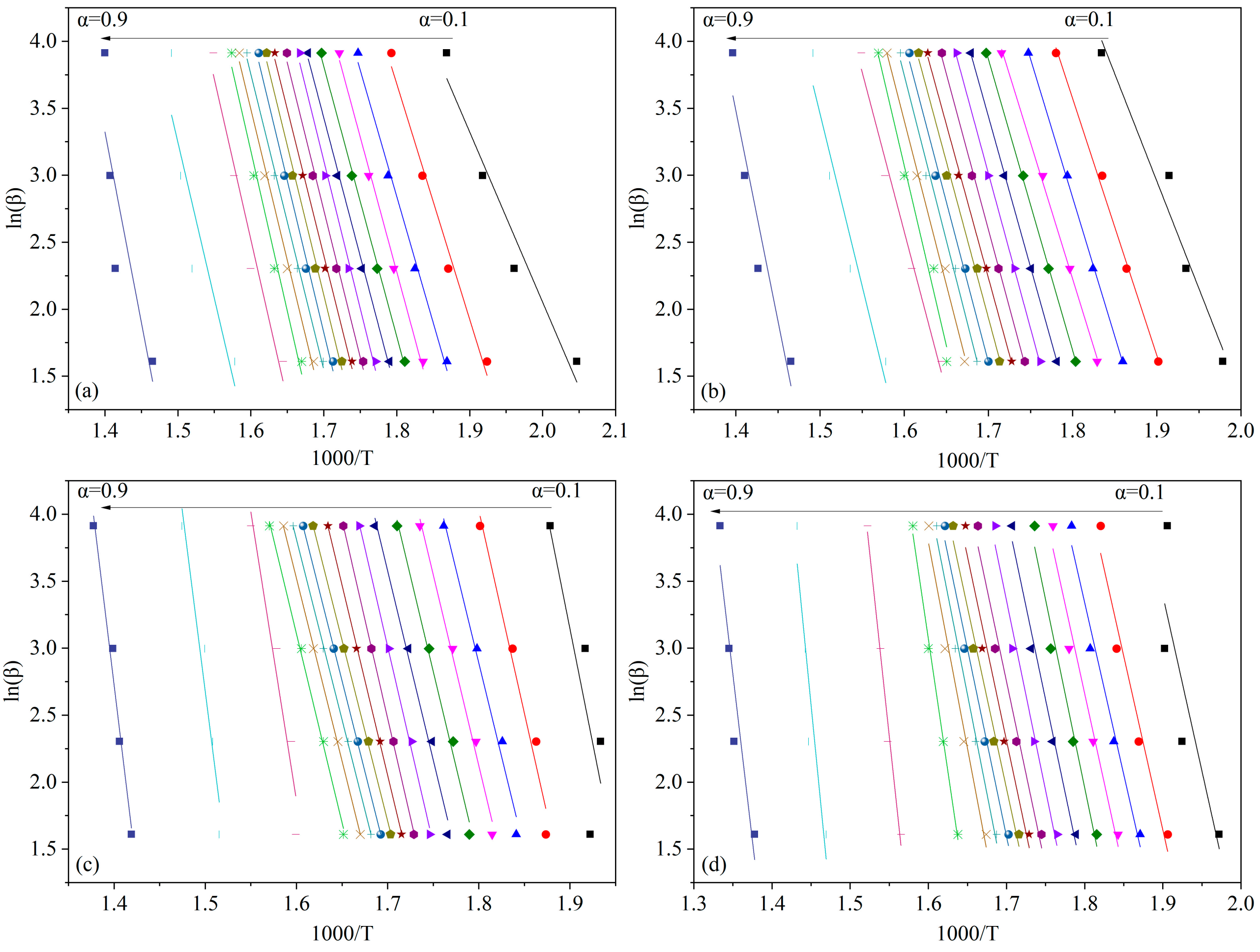


**Figure S3.** Arrhenius plots of FWO method for Cd-contaminated rice straw. (a) RS, (b) RS-0.1%Cd, (c) RS-1%Cd, and (d) RS-5%Cd.

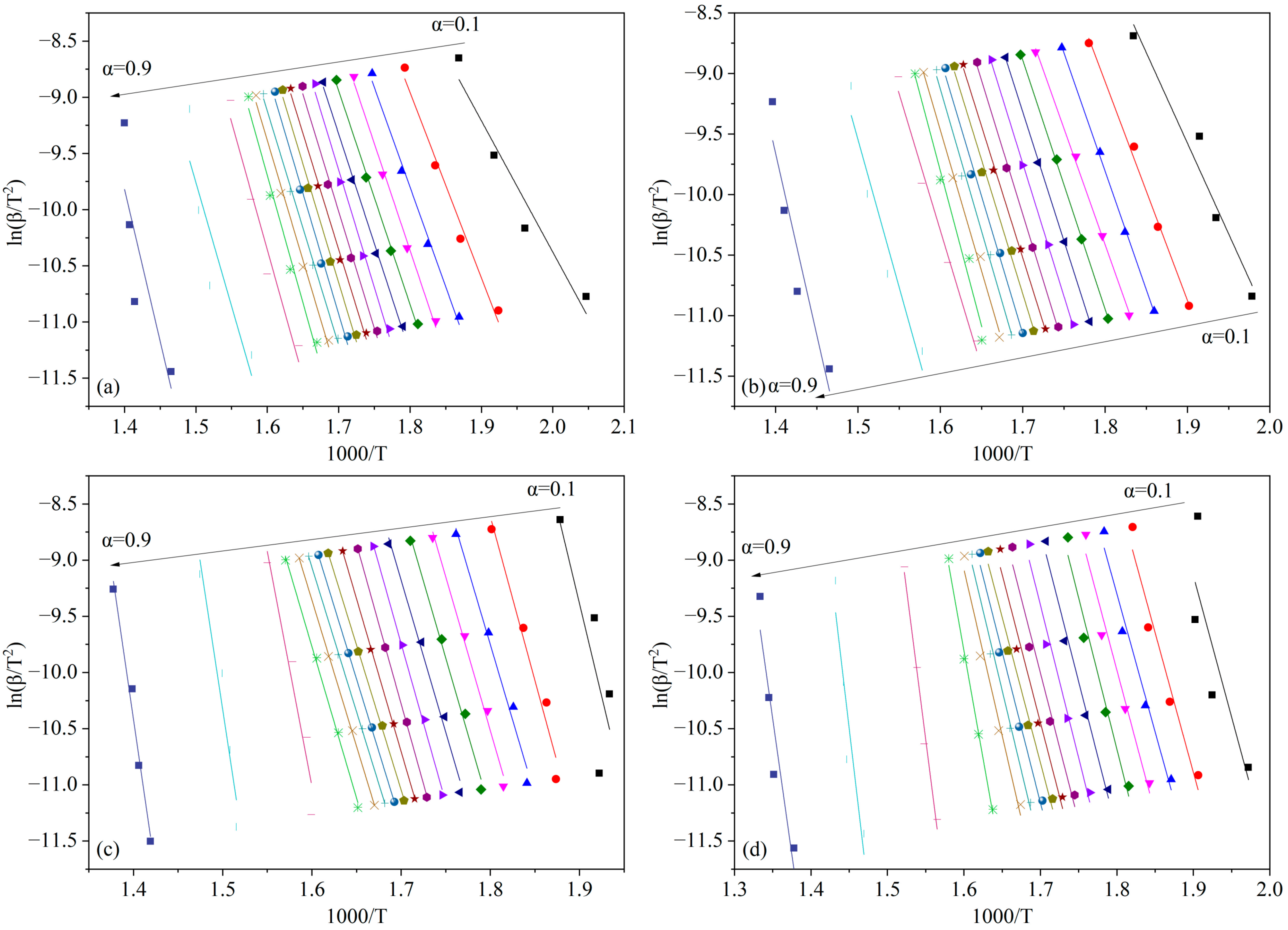


**Figure S4.** Arrhenius plots of KAS method for Cd-contaminated rice straw. (a) RS, (b) RS-0.1%Cd, (c) RS-1%Cd, and (d) RS-5%Cd.
